# Bacterial Seed Endophytes of Domesticated Cucurbits Antagonize Fungal and Oomycete Pathogens Including Powdery Mildew

**DOI:** 10.3389/fmicb.2018.00042

**Published:** 2018-02-05

**Authors:** Eman M. Khalaf, Manish N. Raizada

**Affiliations:** ^1^Department of Plant Agriculture, University of Guelph, Guelph, ON, Canada; ^2^Department of Microbiology and Immunology, Faculty of Pharmacy, Damanhour University, Damanhour, Egypt

**Keywords:** seed endophytes, cucurbit, biocontrol, *Phytophthora capsici*, *Pythium aphanidermatum*, *Rhizoctonia solani*, *Fusarium graminearum*, powdery mildew

## Abstract

The cucurbit vegetables, including cucumbers, melons and pumpkins, have been cultivated for thousands of years without fungicides. However, their seed germination stage is prone to be infected by soil-borne fungal and oomycete pathogens. Endophytes are symbionts that reside inside plant tissues including seeds. Seed endophytes are founders of the juvenile plant microbiome and can promote host defense at seed germination and later stages. We previously isolated 169 bacterial endophytes associated with seeds of diverse cultivated cucurbits. We hypothesized that these endophytes can antagonize major fungal and oomycete pathogens. Here we tested the endophytes for *in vitro* antagonism (dual culture assays) against important soil-borne pathogens (*Rhizoctonia solani*, *Fusarium graminearum*, *Phytophthora capsici*, *Pythium aphanidermatum*). The endophytes were also assayed *in planta* (leaf disk and detached leaf bioassays) for antagonism against a foliar pathogen of global importance, *Podosphaera fuliginea*, the causative agent of cucurbit powdery mildew. The endophytes were further tested *in vitro* for secretion of volatile organic compounds (VOCs) known to induce plant defense. Extracellular ribonuclease activity was also tested, as a subset of pathogenesis-related (PR) proteins of plant hosts implicated in suppression of fungal pathogens, displays ribonuclease activity. An unexpected majority of the endophytes (70%, 118/169) exhibited antagonism to the five phytopathogens, of which 68% (50/73) of *in vitro* antagonists belong to the genera *Bacillus* and *Paenibacillus*. All *Lactococcus* and *Pantoea* endophytes exhibited anti-oomycete activity. However, amongst the most effective inoculants against *Podosphaera fuliginea* were *Pediococcus* and *Pantoea* endophytes. Interestingly, 67% (113/169) of endophytes emitted host defense inducing VOCs (acetoin/diacetyl) and 62% (104/169) secreted extracellular ribonucleases *in vitro*, respectively. These results show that seeds of cultivated cucurbits package microbes with significant disease-suppression potential. As seeds can act as vectors for genetic transmission of endophytes across host generations, it is interesting to hypothesize whether humans, when selecting seeds of healthy hosts, may have inadvertently selected for disease-suppressing seed endophytes. As the majority of pathogen-suppressing endophytes belong to *Bacillus* and *Paenibacillus*, and since *Bacilli* are widely used as commercial biocontrol agents of vegetables, we propose that these agents are mimicking the ecological niche established by their endophytic cousins.

## Introduction

Endophytes including fungi and bacteria are symbionts that colonize the internal tissues of plants without eliciting disease symptoms. They can act as probiotics for their hosts contributing to host fitness and diversity (Ruiza et al., 2011; Hardoim et al., 2015). The endophytic microbiome can improve plant growth and health through four main routes including: (1) protection from biological enemies via production of antimicrobial metabolites and stimulation of plant defense responses; (2) enhancement of host tolerance to abiotic stress; (3) promotion of nutrient acquisition; and (4) secretion of plant growth promoting phytohormones (Berg, 2009; Truyens et al., 2015; Bacon and White, 2016; Khalaf and Raizada, 2016). Beneficial bacteria can emit diverse volatile compounds (VOCs) as signaling molecules to regulate plant growth and immunity in response to biotic and abiotic stresses (Bitas et al., 2013; Asari et al., 2016; Chung et al., 2016). These VOCs, including acetoin and diacetyl, exert their signaling functions by stimulating the transcription of plant host genes employed in metabolic, physiological and defensive activities (Asari et al., 2016). As part of their cascade of defense responses, plants can secrete pathogenesis related proteins (PR proteins), including those with ribonuclease (RNase) activity, that can directly antagonize fungal phytopathogens by penetrating fungal cells or by inducing programmed cell death of plant cells under attack (Filipenko et al., 2013; Miranda et al., 2017; Moosa et al., 2017), though whether endophytes supplement this host activity is not clear.

Beneficial microbes colonizing healthy seeds have the potential to establish the plant microbiome following germination, providing their host plants with nutritional and defensive functions (Johnston-Monje and Raizada, 2011; Truyens et al., 2015; Khalaf and Raizada, 2016; Mitter et al., 2016). Seeds may serve as vectors to transmit endophytes across plant host generations (Hardoim et al., 2015), demonstrated by the relative conservation of seed endophytic diversity across wild plant ancestors and their cultivated domesticates (Johnston-Monje and Raizada, 2011). Though relatively unexploited, seed endophytes are starting to be recognized as promising sources of microbial inoculants, including as biofertilizers (Ruiza et al., 2011; Truyens et al., 2015; Khalaf and Raizada, 2016) but more recently as biocontrol agents (Berg et al., 2017).

**FIGURE 1:**
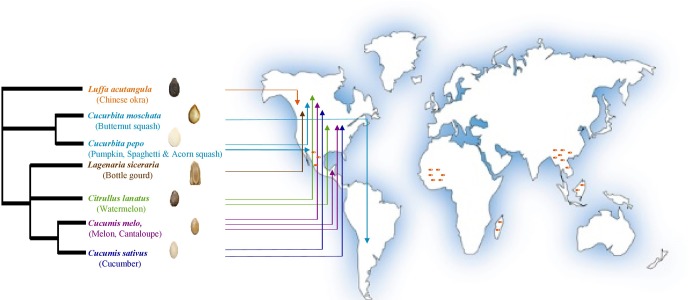
Geographic origins of the cucurbit seeds used in the current study as sources of endophytic bacteria (adapted from Khalaf and Raizada, 2016). Arrows point to the direct geographical origins of seeds and/or fruits. Dark orange dashes refer to the hotspots of cucurbit diversification based on archaeological remains. PhyloT phylogenetic tree online generator was used to construct the phylogenetic tree based on NCBI taxonomic data.

The cucurbit plant family is taxonomically divided into 118–122 genera including 940–980 species that evolutionarily span the world’s continents (Renner et al., 2007; Schaefer et al., 2009; Sebastian et al., 2010; Heneidak and Khalik, 2015; Khalaf and Raizada, 2016; Paris, 2016a). Cucurbits are popular vegetable crops, of which the most economically important globally are cucumber (*Cucumis sativus*), melon (*Cucumis melo*), watermelon (*Citrullus lanatus*), pumpkin and squash (*Cucurbita* sp.). Other cucurbit genera such as *Luffa* and *Lagenaria* are popularly utilized in particular regions of the world (Robinson and Decker-Walters, 1997; Specht et al., 2014; Lebeda et al., 2016; Paris, 2016b). The archaeological remains (mostly seeds) of wild progenitors of major cucurbits reflect their diverse origins spanning the Old World (Africa: watermelon; Asia: melon and cucumber) and the New World (Americas: pumpkins and squash) (**Figure 1**) (Paris, 2016b) including the tropics and humid sub-tropics (Sebastian et al., 2010; Paris, 2016b; Pető et al., 2016; Kates et al., 2017), which are regions that favor oomycete and fungal pathogens.

As with all plants, cucurbit seeds germinate in the soil, and early seed germination is prone to serious soil-derived pathogens that influence seedling survival. Soil pathogens particularly fungi and oomycetes threaten global crop production and food security (Strange and Scott, 2005; Heydari and Pessarakli, 2010; Pliego et al., 2011; Lamichhane et al., 2017). In general, 14.4% of cultivated crops globally are lost every year due to plant diseases with total losses estimated at $220 billion USD (Agrios, 2005; Kandel et al., 2017). Among the most economically important soil-borne oomycetes are *Phytophthora* spp. and *Pythium* spp., while major soil-borne fungal pathogens include *Rhizoctonia solani* and *Fusarium* spp. (Pliego et al., 2011). These soil-borne phytopathogens can infect a wide range of hosts and are able to exist as saprophytes, explaining their ability to spread through soil even in the absence of a host plant (Lamichhane et al., 2017). Today, there is interest in understanding the interactions between the plant microbiome and oomycetes as a major step toward protecting plants against oomycetous infections (Larousse and Galiana, 2017). Although the oomycete *Phytopthora capsici* was first identified as a chili pepper pathogen in Mexico in 1922, it was subsequently discovered to be a devastating vegetable crop pathogen infecting numerous species of plants in different plant families such as the Solanaceae (e.g., potato, tomato) and cucurbit families (Hausbeck and Lamour, 2004). *Pythium* sp. enter cucumber roots through the root hairs (Yang et al., 2004), and within this genus, *Pythium aphanidermatum* causes dramatic losses in greenhouse cucumbers in particular when using hydroponic systems due to the resulting root rot (Punja and Yip, 2003). Among the fungal pathogens of crops, *R. solani* is devastating and difficult to manage as the fungus develops sclerotia and is difficult to control chemically. The fungus is normally found in low densities in the soil as a saprotrophic and facultative parasite that enables it to infect a broad spectrum of plant species causing serious losses to trees, major grain and vegetable crops including cucurbits, in particular cucumber in both field and greenhouse production (Val-Moraes, 2015; Karimi et al., 2016; Justyna et al., 2017). *Fusarium* spp. such as *F. oxysporum* are known to cause root rot in cucurbits, however *F. graminearum* is ranked fourth among the top 10 most severe fungal pathogens of crops based on voting by the international research community (Dean et al., 2012). Biocontrol of *F. graminearum* in cereals is of global interest due to its serious detrimental effect of reducing the grain quality and producing mycotoxins (Mousa et al., 2016).

At later stages following germination, plants are vulnerable to airborne pathogens. In cucurbits, powdery mildew is an airborne foliar disease of global importance in field and greenhouse production, leading to serious yield losses and poor quality fruits. Infection frequently occurs by two air-borne obligate pathogens, *Golovinomyces orontii* and *Podosphaera xanthii* (Braun and Cook, 2012; Lebeda et al., 2016). *Podosphaera xanthii*, previously known as *Sphaerotheca fusca* or *Sphaerotheca fulginiea*, is frequently reported in warmer climates such as subtropical and tropical regions and under greenhouse conditions (Cohen, 1993; Shishkoff, 2000; Lebeda et al., 2016).

Primary screening of potentially beneficial microbes for antagonism of culturable phytopathogens is typically performed using the *in vitro* dual culture assay where the pathogen target and the candidate biocontrol agents are cultured together to assay for direct pathogen growth suppression (Pliego et al., 2011; Bosmans et al., 2016). This method has been shown to predict their interactions *in planta* (Shehata et al., 2016). As this option is not available for obligate pathogens such as *P. xanthii*, leaf disk and detached plant organ assays are used where the pathogen is maintained on plant tissues in Petri dishes supplemented with water and nutrients. These *in planta* assays have been used to screen for candidates as biocontrol agents and their extracts against powdery mildew in cucumber (Cohen, 1993; Lebeda et al., 2016).

We previously reported an extensive collection of seed associated bacterial endophytes from cultivated cucurbit crops along with their exhibited plant growth promoting activity but we did not test them for potential biocontrol traits (Khalaf and Raizada, 2016). Given the cultivation of cucurbits over thousands of years in warm, moist environments, including more recently in greenhouses, here we used the endophyte library to test the hypothesis that endophytes associated with seeds of the world’s human-selected cucurbits have the ability to restrain soil-borne oomycete and fungal pathogens (the culturable pathogens, *P. capsici*, *P. aphanidermatum*, *R. solani* and *F. graminearum*) known to infect cucurbits and other plants following seed germination. We also tested whether the endophytes can suppress the later cucumber foliar disease, powdery mildew, caused by the airborne fungal pathogen *Podosphaera xanthii.* For the culturable pathogens, the *in vitro* dual culture assay was used. For the obligate pathogen, the leaf disk and detached whole leaf assays were used. To test for the potential ability of the endophytes to stimulate host defense responses, they were *in vitro* screened for secretion of the VOC compounds, acetoin and diacetyl. The endophyte library was also screened for extracellular ribonuclease activity.

## Results

### Predominance of Endophytic Functional Traits Known to Promote Plant Disease Resistance

**FIGURE 2:**
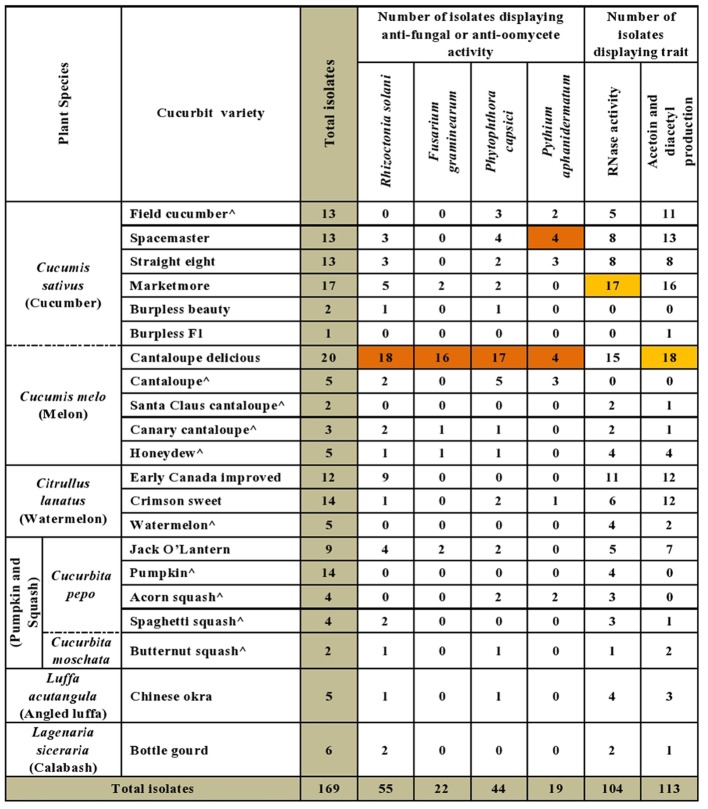
Summary of *in vitro* antagonistic and functional activities displayed by the endophytic bacteria associated with cucurbit seeds, organized by host taxonomy. Each orange highlight denotes the crop variety that possesses the greatest number of endophytes that display the corresponding beneficial functional trait (vertical column) *in vitro.* The circumflex accent (ˆ) denotes that fresh fruits were used as the seed source.

**FIGURE 3:**
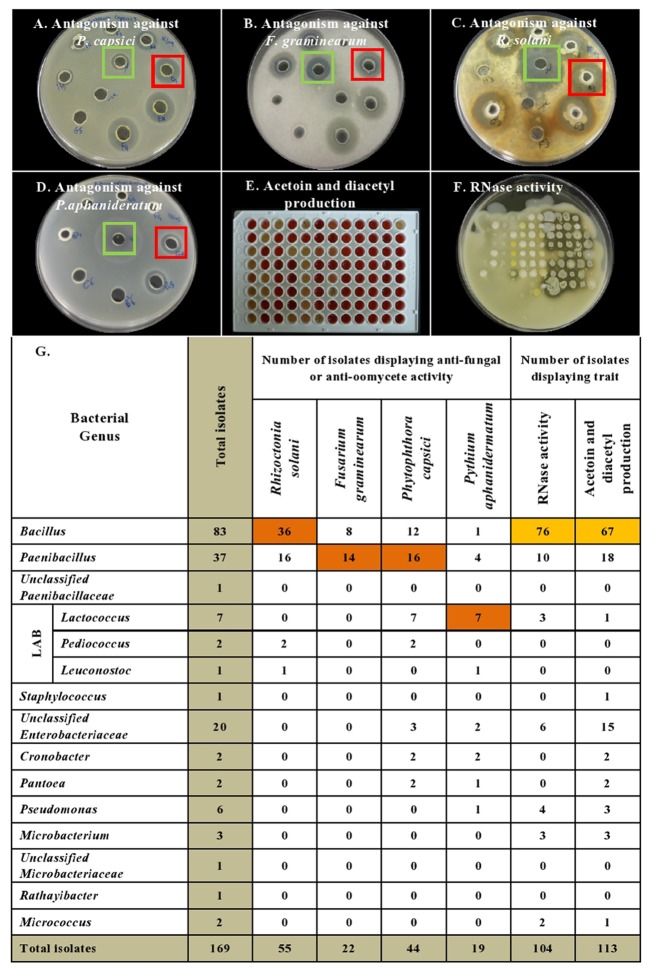
Summary of *in vitro* antagonistic and functional activities displayed by the endophytic bacteria associated with cucurbit seeds, organized by bacterial taxonomy. **(A–D)** Examples of endophytes that antagonize the growth of pathogens *in vitro* using the disk diffusion technique. Green squares indicate positive controls (fungicide), while red squares indicate antagonistic endophytes. Shown are antagonism assays for: **(A)** *Phytophthora capsici*, **(B)** *Fusarium graminearum*, **(C)** *Rhizoctonia solani*, and **(D)** *Pythium aphanidermatum*. **(E,F)** Examples of endophytes that display functional traits known to promote host plant defense, specifically: **(E)** Acetoin and/or diacetyl production (red/pink is a positive result), and **(F)** RNase activity (clear halo around colony is a positive result). **(G)** Summary of the bacterial endophytes that display the tested *in vitro* activities. Each orange highlight denotes the bacterial genus that possesses the greatest number of endophytes that display the corresponding beneficial functional trait (vertical column) *in vitro.*

A majority of the cucurbit seed associated endophytes expressed functional traits known to enhance plant disease resistance. Approximately 67% (113/169) and 62% (104/169) of the tested endophytic bacteria produced acetoin and/or diacetyl *in vitro* and displayed ribonuclease (RNase) activity, respectively (**Figures 2**, **3** and **Supplementary Table S1**). Cucumber seeds, specifically, were the primary source of the positive candidates accounting for 44% (49/113) of the acetoin/diacetyl producers and 37% (38/104) of strains with RNase activity. The genus *Bacillus* accounted for 59% (67/113) of acetoin/diacetyl producers and 73% (76/104) of RNase positive isolates. Furthermore, the vast majority of the 83 *Bacillus* isolates displayed acetoin/diacetyl activity (81%, 67/83) and RNAse activity (92%, 76/83) (**Figure 3**). In addition, 49% (18/37) and 27% (10/37) of the *Paenibacillus* isolates demonstrated acetoin/diacetyl production and RNase activity *in vitro*, respectively. Regarding the identified *Enterobacteriaceae* family members, 75% (15/20) were acetoin/ diacetyl producers and 30% (6/20) showed RNase activity. All isolated bacterial endophytes of the genera *Cronobacter*, *Pantoea*, *Microbacterium*, and *Staphylococcus* displayed these traits.

### Predominance of *in Vitro* Anti-pathogenic Activity of Cultivated Cucurbit Seed-Associated Endophytes

The bacterial endophytes were tested for their ability to suppress growth *in vitro* of a spectrum of plant pathogens with diverse host ranges including cucurbits. In total, 43% (73/169) of isolates showed anti-pathogenic traits *in vitro* including against both oomycete pathogens (*P. capsici*, *P. aphanidermatum*) and fungal pathogens (*R. solani*, *F. graminearum*) of which *F. graminearum* (control) is not known to cause disease in cucurbits (**Figure 2**). For each pathogen tested, antagonistic endophytes were identified that originated from diverse, major cucurbits. For example, the potential *R. solani* antagonists originated from the five cucurbit genera and 15 different varieties. The seeds of diverse varieties of melon (*C. melo*), in particular variety Cantaloupe delicious (**Figure 2** and **Supplementary Table S1**), hosted the highest number and broadest spectrum (fungal, oomycetes) of *in-vitro* antagonistic endophytic bacteria. For example, 82% (18/22) of cucurbit family endophytes that displayed anti-*F. graminearum* activity originated from melon seed; this number was 55% (24/44) for *P. capsici* and 42% (23/55) for *R. solani* (**Supplementary Table S1**). Despite desiccation and long-term storage, dry seeds (variety names without asterisks, **Figure 2** and **Supplementary Table S1**) were the main supplier of bacterial endophytes in this study (113/169) and constituted the majority of the antagonists (77%, 56/73 of antagonists). Fresh seeds were also sources of antagonists, however, (e.g., melon, *C. melo*; **Figure 2** and **Supplementary Table S1**).

With respect to the tested fungal pathogens, of the 55 strains that antagonized *R. solani in vitro*, 52 belonged to the genera *Bacillus* and *Paenibacillus* though they only constituted 71% (120/169) of the endophyte library (**Figure 3** and **Supplementary Table S1**). Similarly, of the 22 strains that antagonized *F. graminearum*, all belonged to *Bacillus* and *Paenibacillus.* By contrast of the 20 *Enterobacteriaceae* endophytes tested, none suppressed the fungal pathogens (**Figure 3** and **Supplementary Table S1**).

A broader taxonomic spectrum of endophytes suppressed growth of the tested oomycete pathogens. Nevertheless, of the 44 endophytes that suppressed growth of *P. capsici in vitro*, 64% (28/44) belonged to *Bacillus* and *Paenibacillus*, with the remainder belonging to 5 other genera including *Lactococcus* (16%, 7/44). There were 19 taxonomically diverse strains that suppressed *P. aphanidermatum in vitro*, of which *Lactococcus* endophytes were the most prevalent (41%, 7/19), whereas only 5 *Bacillus* and *Paenibacillus* strains (combined) were antagonists. It is noteworthy that all 7 isolates of the genus *Lactococcus* showed *in vitro* anti-oomycete activity (**Figure 3** and **Supplementary Table S1**).

### Leaf Disk Bioassay for Antagonism against Cucumber Powdery Mildew

**FIGURE 4:**
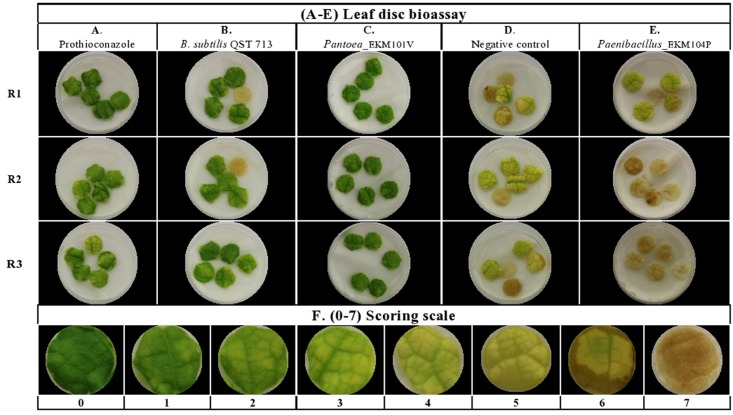
Screening of cucurbit seed associated endophytic bacteria for antagonism against cucumber powdery mildew disease using a leaf disk bioassay. **(A–E)** Representative pictures of: **(A)** chemical fungicide positive control (Prothioconazole), **(B)** commercial biocontrol agent positive control (*Bacillus subtilis* strain QST 713), **(C)** example of a typical promising endophytic bacteria; *Pantoea* EKM101V, **(D)** the negative control (LB broth amended with filter sterilized 0.01% Tween 20) and **(E)** example of an endophytic bacteria displaying adverse effects in the presence of the pathogen. *Paenibacillus* EKM104P. **(F)** Represents pictures of 0–7 scoring scale used as references to guide the visual disease assessments. R (1–3) denotes replicate number.

**FIGURE 5:**
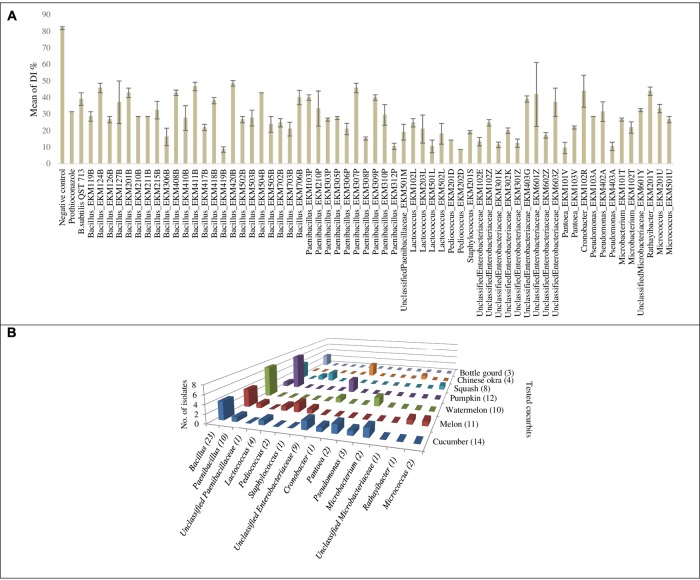
Graphical representation for endophytic bacteria that exhibited significant suppression of powdery mildew in the leaf disk bioassay: **(A)** Graphical representation of calculated means of the disease index percentage (DI%) displayed by controls and endophytic bacteria that exhibited significant suppression of powdery mildew (*p* < 0.05) in the leaf disk bioassay. Error bars show the standard error of mean (SEM). **(B)** Graph chart summarizing endophytic bacterial genera that exhibited significant suppression of powdery mildew symptoms at *p* < 0.05, cross referenced with the original endophyte seed sources. Numbers between brackets displayed on the horizontal axis denote the total number of strains in each corresponding bacterial genus that showed antagonism. Numbers between brackets displayed on the depth axis denote the total number of strains identified from each corresponding cucurbit crop that showed antagonism. For detailed information by endophyte strain, see **Supplementary Tables S3**, **S4**.

The endophytes were also tested for antagonism against *Podosphaera fuliginea*, the causative agent of cucumber powdery mildew disease. As the pathogen is obligate, the bioassay employed leaf disks (**Figures 4A–D**). The disease index (DI%) and preventive effect (PE%) were calculated for all tested endophytes (**Supplementary Tables S2B**,**C**). In total, 37% (62/169) of the tested endophytic bacteria demonstrated significant suppression of powdery mildew symptoms (*p* < 0.05, mean DI%) in comparison to disks treated with LB media (negative control) (**Figures 4D**, **5A** and **Supplementary Tables S3**, **S4**). Noteworthy, these promising 62 strains originated from the five tested cucurbit genera (**Figure 5B** and **Supplementary Table S2A**). Interestingly, most of the remaining endophytes displayed adverse effects on cucumber leaf health in the presence of the pathogen (**Figure 4E** and **Supplementary Tables S2A**–**C**). The beneficial endophytes displayed mean positive PE values of up to 89%. Of these beneficial strains, 66% (41/62) and 79% (49/62) were statistically equal or more effective (One-Way ANOVA Dunnett’s multiple comparisons) than the fungicide, prothioconazole [(positive control, concentration optimized in a pre-experiment (see Materials and Methods; data not shown)] (**Figure 4A**) and the biofungicide, *Bacillus subtilis* strain QST 713 (**Figure 4B**), respectively.

Unlike *in vitro* antimicrobial activity, fresh cucurbit seeds were the major sources (61%, 38/62) of antagonists that significantly suppressed powdery mildew *in planta* (**Supplementary Tables S2A**, **S3**). Furthermore, cucumber, pumpkin, cantaloupe and watermelon seeds contributed many of the antagonists, accounting for 23% (14/62), 19% (12/62), 18% (11/62) and 16% (10/62) of the significantly (*p* < 0.05) effective endophytes, respectively. With respect to endophytic taxonomy, the strains that significantly restrained symptoms of the disease belonged to 14 different bacterial genera (**Figure 5B** and **Supplementary Tables S3**, **S4**), of which the most prevalent (in rank order) were *Bacillus* (37%, 23/62), *Paenibacillus* (16%, 10/62) and *Unclassified Enterobacteriaceae* (14.5%, 9/62). However, amongst the most effective antagonists were *Pediococcus* and *Pantoea* endophytes (**Figure 4C**).

### Detached Whole Leaf Bioassay for Antagonism against Cucumber Powdery Mildew

**FIGURE 6:**
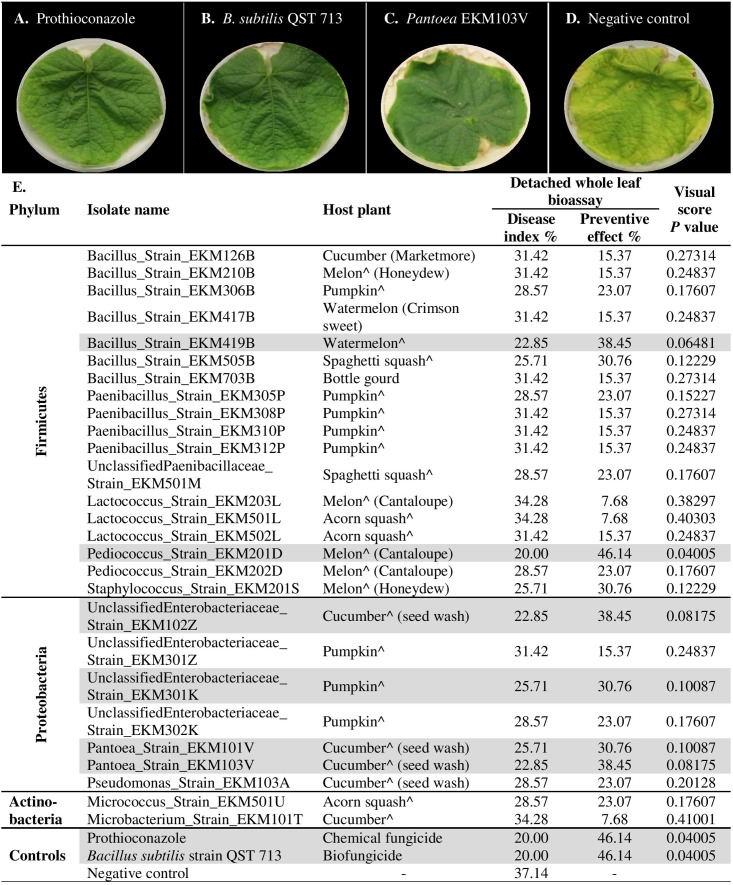
*In planta* screening of promising endophytes from the leaf disk bioassay for antagonism against cucumber powdery mildew using a detached whole leaf bioassay. **(A–D)** Representative pictures of: **(A)** chemical fungicide positive control (Prothioconazole), **(B)** commercial biocontrol agent positive control (*Bacillus subtilis* strain QST 713), **(C)** example of a typical promising endophytic bacteria; *Pantoea* EKM103V, **(D)** and the negative control (LB broth amended with filter sterilized 0.01% Tween 20). **(E)** List of tested endophytic bacterial strains, their respective plant seed source, corresponding disease index, preventive effect values and visual score *p*-values (calculated using independent samples *t*-test, one-tailed and unequal variance). The rows highlighted in gray denote promising strains that significantly (*p* < 0.1) suppressed the disease in comparison to the negative controls. A greater Disease Index indicates increased disease symptoms, whereas a greater Preventative Effect denotes improved control of the disease. The circumflex accent (ˆ) denotes that fresh fruits were used as the seed source.

To independently validate the leaf disk results and to make the assay more robust, the endophytes that were effective against powdery mildew in comparison to prothioconazole fungicide (41 strains, **Supplementary Table S2D**) were re-tested using detached whole cucumber leaves infected with the pathogen (**Figures 6A–E** and **Supplementary Figure S1**). In total, 66% (27/41) of endophytes were confirmed to inhibit powdery mildew symptoms visually in comparison to leaves treated with LB media (negative control), with PE values ranging from 8–46% (**Figure 6E**, **Supplementary Figures S1A–E**, and **Supplementary Table S2D**). However, only six strains showed significant suppression of powdery mildew disease when compared to the negative control (independent *t*-test, one-tailed, unequal variance, using visual scores at *p* < 0.1) (**Figure 6E** and **Supplementary Figure S1**). As above, the majority of the remaining endophytes displayed adverse effects on cucumber leaf health in the presence of the pathogen (**Supplementary Figure S1E** and **Supplementary Table S2D**). The fresh seeds were the major sources of beneficial microbes, accounting for 89% (24/27) of disease antagonists (**Figure 6E**). Pumpkin seeds were the main source of promising antagonists accounting for 30% (8/27) of candidates, followed by cucumber seeds 22% (6/27), cantaloupe (19%, 5/27) and watermelon seeds (19%, 5/27). Nevertheless, 50% (3/6) of strains that exhibited significant disease suppression originated from fresh cucumber seeds and belong to the *Enterobacteriaceae* family (**Figure 6E**). *Bacillus* endophytes were the most prevalent antagonists, comprising 26% (7/27) of the positive strains. However, a *Pediococcus* endophyte from fresh cantaloupe seeds displayed the highest PE% against the disease (46%), equivalent to the positive controls (**Supplementary Table S2D**).

## Discussion

The *Cucurbitaceae* is an economically important crop family, associated with human civilizations for thousands of years (Jeffrey, 1980) and able to grow in diverse ecosystems and climates around the world (**Figure 1**) (Pessarakli, 2016). These crops originated and were domesticated in the tropics and humid sub-tropics across the world’s continents, and today also grow in warm, moist greenhouses (Sebastian et al., 2010; Paris, 2016b; Pető et al., 2016; Kates et al., 2017); these environments favor fungal pathogens. Unlike endophytes that inhabit other plant organs, seed endophytes have the potential to transmit across plant generations and are uniquely able to establish the plant microbiome in seedlings (Johnston-Monje and Raizada, 2011; Truyens et al., 2015; Huang et al., 2016; Johnston-Monje et al., 2016; Khalaf and Raizada, 2016; Mitter et al., 2016). Previous studies have shown that up to one third of *in vitro* tested endophytes exhibit activity against plant pathogens (Berg, 2009). For these reasons, we hypothesized that the seeds of domesticated cucurbits may possess endophytes with anti-pathogen traits. We previously isolated cucurbit seed-associated endophytes and showed that they express plant growth promoting traits including nutrient acquisition and phytohormone biosynthesis (Khalaf and Raizada, 2016).

Here we tested the seed associated endophytes against the most important soil-borne pathogenic genera of crops (*Fusarium*, *Rhizoctonia, Pythium* and *Phytophthora*) known to be detrimental to early seed germination (Nelson, 2017), along with the most devastating foliar disease of cucurbits, powdery mildew (Glawe, 2008). Our findings showed that a surprisingly large number of seed-associated endophytes (70%, 118/169) could antagonize the five tested phytopathogens. when agar disk assay results (4 pathogens, 73/118) were combined with a detached leaf disk bioassay (one obligate pathogen, 45/118). Furthermore, approximately two-thirds of the library exhibited RNase activity and secrete the VOCs, acetoin and/or diacetyl, known to be implicated in triggering the plant immune defense. Combined, the results show that the majority of the seed associated bacteriome of the domesticated cucurbits possess potential biocontrol traits.

### Importance of *Bacillus* and *Paenibacillus* Seed Endophytes

*Bacillus* and *Paenibacillus* are cosmopolitan aerobic bacterial genera in agroecosystems (McSpadden Gardener, 2004). Their ubiquitous existence has been confirmed by culture dependent techniques, while metagenomic analyses have revealed additional species diversity (Borneman et al., 1996; Mahaffee and Kloepper, 1997; Garbeva et al., 2003). Moreover, they are known for specific attributes that favor them as crop inoculants including their ability: (1) to form stress tolerant endospores; (2) to secrete diverse antimicrobial secondary metabolites (peptide antibiotics); (3) to secrete extracellular enzymes and signaling molecules; (4) to scavenge nutrients from the environment; (5) to biosynthesize phytohormones; and (6) to induce host systemic defense. Various beneficial strains of *Bacillus* and *Paenibacillus* are commercially available as biopesticides and biofertilizers (McSpadden Gardener and Fravel, 2002; McSpadden Gardener, 2004; Choudhary and Johri, 2009; Böhme et al., 2016).

Despite an earlier focus of biocontrol research on the use of Gram negative bacteria such as *Pseudomonas*, *Agrobacterium* and *Erwinia*, later studies starting in 1999 introduced *Bacillus* species as alternative biocontrol agents (Emmert and Handelsman, 1999; Nagórska et al., 2007; Rybakova et al., 2016). The above-noted biological traits of *Bacilli* facilitated their commercial formulation as marketable biopesticides (Ongena and Jacques, 2008; Rybakova et al., 2016). For example, in a recent study (Karimi et al., 2016), 4/8 *Bacillus* strains (including *B. amyloliquefaciens*, *B. pumilus* and *B. siamensis*) isolated from the sugar beet rhizosphere, and shoots and roots of apple, showed significant suppression of pathogenic *R. solani* infecting sugar beet under greenhouse conditions.

Interestingly, here, 43% (36/83) of *Bacillus* strains isolated from cucurbit seeds exhibited antagonism *in vitro* against diverse fungal and oomycete pathogens including *R. solani* (isolated from sugar beet), *F. graminearum* (from maize), *P. capsici* (from pepper) and *P. aphanidermatum* (from cucumber). These *Bacillus* isolates originated from seeds of all tested cucurbit species (**Figure 2**) and were predicted to be diverse species (Khalaf and Raizada, 2016). In a previous study, various *Bacillus* strains (*B. subtilis, B. amyloliquefaciens*, and *B. cereus*) isolated from cucurbit fruits (but not seeds) (*C. melo, C. callosus*, *Citrullus lanatus* and *Bryonia cretica*) demonstrated *in vitro* anatagonism against four fungal pathogens of melons including *F. oxysporum F. sp. melonis* and *F. oxysporum, and F. sp. radicis-cucumerinum* (Glassner et al., 2015). Furthermore, 71% of tested *Bacillus* microbes against Fusarium wilt in cucumber were effective (Raza et al., 2017). Fusarium wilt disease is a devastating disease of cucumber, caused by *Fusarium oxysporum* (Raza et al., 2017). We do not know if the anti-*F. graminearum* strains identified in this study showed cross-antagonism toward other *Fusarium* species, but it may explain the reason why cucurbits possess endophytes that antagonize a non-cucurbit pathogen.

With regard to the role of *Bacillus* in managing cucurbit powdery mildew, in a previous study, four *Bacillus* strains isolated from the healthy phyllosphere and rhizosphere of cucurbits infected with powdery mildew demonstrated antagonism against the fungus *Podosphaera fusca* on detached melon leaves and seedlings; the suppressive efficacy was up to 80% (Romero et al., 2004). In another study, *Bacillus subtilis* strain UMAF6639 demonstrated reduction of powdery mildew disease severity in infected melon leaves (García-Gutiérrez et al., 2012). The antagonism involved three major mechanisms including induction of host systemic resistance (García-Gutiérrez et al., 2012), stimulation of plant signaling pathways mediated by jasmonate and salicylic acid, and finally induction of host defense mediated by a surfactin lipopeptide. Generally, however, powdery mildew antagonism by biocontrol agents has been shown to be caused by microbial production of the antifungal compounds, iturin and fengycin (García-Gutiérrez et al., 2013).

Since the first characterization of the genus *Paenibacillus* 20 years ago, numerous studies of its role as an endophyte have been reported (Rybakova et al., 2016). For example, *P. polymyxa* strains identified in our lab from seeds and roots of maize exhibited anti-*F. graminearum* activity *in vitro* as well as under greenhouse conditions (Mousa et al., 2015). The displayed antagonism was confirmed by molecular detection of the *fus*A gene and biochemical detection of produced fusaricidin derivatives. Similarly, 3 out of 4 isolates belonging to the genus *Paenibacillus* (and one isolate belonging to the genus *Pantoea*) identified from wheat seeds of a commercial variety exhibited anti-*F. graminearum* activity in the dual culture assay (Díaz Herrera et al., 2016). Moreover, a *P. polymyxa* strain isolated from the vinegar industry showed effective suppression of Fusarium wilt in cucumber and promoted other beneficial microbes to successfully compete against the causative agent (*F. oxysporum*, a soil pathogen) for colonizing the rhizospheric niche of cucumber (Shi et al., 2017). *P. polymyxa* PB71 identified from seeds of Styrian oil pumpkin demonstrated antagonism against fungal diseases caused by *Didymella bryoniae* under greenhouse condition as well as suppression of powdery mildew during field trials, and improved pumpkin yield (Fürnkranz et al., 2012). In a recent study (Liu et al., 2017), endophytic bacterial strain RSE1, isolated from rice (*Oryza sativa L*.) seeds and identified as *P. polymyxa*, showed *in vitro* antagonism against the fungal pathogen *Ustilaginoidea oryzae*, the causative agent of rice false smut. The genetic basis of the biocontrol trait was predicted through computational analysis of the draft genome of the endophytic strain as a glucanase gene. In our study, 17/37 (46%) of *Paenibacillus* strains isolated from commercial cucurbit seeds exhibited *in vitro* antagonism when results of all tested crop pathogens were combined (**Supplementary Table S1**). These candidates included 16 antagonists of *R. solani* and *P. capsici*, and 14 strains against *F. graminearum in vitro* (**Figure 3**). Intriguingly, the *R. solani* antagonists were isolated from all tested cucurbit genera and hence may have been inadvertently selected. Almost all of these promising candidates were acetoin and/or diacetyl producers (**Supplementary Table S1**). Seven strains of *Paenibacillus* recovered from fresh seeds of pumpkin showed significant reduction of powdery mildew symptoms (*p* < 0.01–0.0001) in the leaf disk bioassay (**Supplementary Tables S3**, **S4**). Our results are congruent with the reported patents of *Paenibacillus* species as biocontrol agents antagonizing a wide range of plant pathogens comprising pathogenic species of *Fusarium*, *Rhizoctonia*, *Pythium*, *Phytophthora*, *Botrytis*, *Penicillium*, *Sclerotinia* and *Cladosporium* (Rybakova et al., 2016).

### Importance of Other Endophytic Genera

The genus *Pantoea* was first reported in the literature as a plant pathogen (Muraschi et al., 1965; Nadarasah and Stavrinides, 2014), but more recently various strains have been shown to exert beneficial biocontrol activity to host plants through various mechanisms including competitive colonization, production of antibiotics and/or induction of host systemic defense. *Pantoea* species have been shown to target a wide spectrum of plant pathogens including bacteria, fungi, oomycetes and parasitic nematodes via secretion of antimicrobials such as pantocins, herbicolins, microcins, and phenazines (Walterson and Stavrinides, 2015). Commercially formulated *Pantoea*-based biopesticides are currently available such as BlightBan C9-1 (*P. vagans* C9-1) and Bloomtime Biological (*P. agglomerans* E325) which are used for combating fire blight in apples and pears (Johnson and Stockwell, 1998; Pusey, 2002). In a recently published paper, three endophytic bacteria isolated from surface-sterilized rice (*Oryza sativa L*.) seeds promoted *in vitro* rice seedling development along with protection against *F. oxysporum*. Using 16S rRNA sequencing, the strains were identified as *Pantoea dispersa*, along with *Enterobacter asburiae* and *Pseudomonas putida* (Verma et al., 2017). In our study, we isolated two *Pantoea* strains from fresh cucumber seed mucilage that exhibited *in vitro* antagonism against the tested oomycetes (**Supplementary Table S1**) and showed significant suppression of powdery mildew symptoms in the leaf disk bioassay (*p* < 0.05) and detached whole leaf bioassay (*p* < 0.1) (**Figures 4C**, **6C** and **Supplementary Tables S2**, **S3**). These strains were also producers of acetoin and diacetyl (**Supplementary Table S1**). Our findings suggest that *Pantoea* may hold potential as inoculants that can target a new, wider anti-pathogenic spectrum.

Another recent study (Sandhya et al., 2017) showed the potential of seed endophytes as biocontrol agents. In that study, maize seeds and roots were used as sources of bacterial endophytes; 10 out of 39 endophytic bacteria demonstrated diverse *in vitro* activities comprising growth promotion and antagonism against 6 fungal phytopathogens (*Macrophomina phaseolina*, *Rhizoctonia solani*, *Fusarium oxysporum*, *Sclerotium rolfsii*, *Pythium aphanidermatum* and *Alternaria* sp.) under drought stress. These endophytic bacteria were identified to the genus level as *Pseudomonas*, *Acinetobacter*, *Enterobacter*, and *Sinorhizobium.*

In the food industry, various species of lactic acid bacteria (LAB) belonging to the genera *Enterococcus, Lactobacillus, Lactococcus, Pediococcus* and *Leuconostoc*, are well known safe biopreservatives and biological control agents (Gajbhiye and Kapadnis, 2016; Porto et al., 2017). Their antagonism is mediated by secretion of cyclic dipeptides, diacetyl, organic aids, ethanol, antifungal metabolites (such as phenyllactic acid or fatty acids), bacteriocins and antibiotics (such as reutericyclin) (Akbar et al., 2016; Gajbhiye and Kapadnis, 2016). In our study, the results revealed 10 antifungal LAB strains belonging to the genera *Lactococcus, Pediococcus* and *Leuconostoc.* All *Lactococcus* strains showed *in vitro* antagonism against oomycetes (**Supplementary Table S1**) whereas four strains antagonized *Podosphaera fuliginea in planta* (**Supplementary Table S2A**). The two identified *Pediococcus* strains were shown here to exhibit a broad antimicrobial target spectrum including against *R. solani*, *P. capsici* and *Podosphaera fuliginea*, but not *F. graminearum.* However, in a previous study, a *Pediococcus pentosaceus* strain isolated from dairy products antagonized *F. graminearum* growth (Sellamani et al., 2016). Moreover, the broad antifungal spectrum of *Pediococcus acidilactici* LAB 5 strain was first reported in a study by Mandal et al. (2007) and included antagonism against *Alternaria solani*, *Aspergillus fumigaus*, *A. parasiticus*, *Cladosporium herbarum*, *Colletotrichum acutatum*, *Curvularia lunata*, *Fusarium oxysporum*, *Microsporium* sp. *Mucor* sp. and *Penicillium* sp. (Mandal et al., 2007). In this study, a single *Leuconostoc* strain demonstrated antagonism toward *R. solani* and *P. aphanidermatum*, though in an earlier study, the intimate association between *R. solani* and *Leuconostoc* bacteria in sugar beet roots was interpreted as a synergistic interaction that promoted root rot (Strausbaugh, 2015). Combining these previous reports with our study, it would appear that the probiotic characteristics of LAB are not limited to food preservation, but may extend to counteract plant pathogens during crop cultivation.

### Acetoin and Diacetyl Production

As noted earlier, acetoin (3-hydroxy-2-butanone) and its major bioanalogs (2,3-butanediol and diacetyl), are important volatile organic compounds (VOCs) produced by several microbes including bacteria. In earlier research, *B. subtilis*, *B. amyloliquefaciens* and *P. polymyxa* demonstrated growth promotion and induction of systemic resistance in *Arabidopsis thaliana* by secretion of these VOCs (Ryu et al., 2003, 2004; Lee et al., 2012). In our study, perhaps surprisingly, production of acetoin and diacetyl volatiles correlated to antagonism of fungi and oomycetes despite the lack of a plant host in our *in vitro* assay. For example, 95% (21/22), 82% (45/55), 77% (34/44) and 58% (11/19), respectively, of *F. graminearum*, *R. solani*, *P. capsici* and *P. aphanidermatum in vitro* antagonists, were acetoin and diacetyl producers (**Supplementary Table S1**). Furthermore, 43.5% (27/62) of promising candidates that significantly (*p* < 0.05) reduced disease severity against powdery mildew in the *in planta* leaf disk bioassay were VOC producers (**Supplementary Table S2**). It may be that these endophytes have been selected to have multiple anti-pathogen mechanisms, including both direct antibiosis (evident in our dual-culture assays) and induction of host defense. The majority of the promising VOC-producing antagonists in this study belong to the phylum *Firmicutes*, in particular the genera *Bacillus* and *Paenibacillus* (**Figure 3**). Similarly, in an earlier study, four different VOC-producing *B. amyloliquefaciens* subspecies demonstrated *in vitro* antagonism against several plant pathogenic fungi (Asari et al., 2016). Therefore, our findings point to the promise of using endophytic bacteria displaying VOC activity in biological control applications (Vespermann et al., 2007; Asari et al., 2016), though further confirmation of the impact of the VOCs produced by the cucurbit endophytes will be required. Specifically, VOCs of promising biocontrol candidates could be collected in sealed plates and applied to the plant tissue in the presence of the relevant pathogen.

### Role of Ribonuclease Activity in Biocontrol

In our study, of the 104 RNase positive isolates, 91% (20/22) of *F. graminearum* antagonists displayed RNase activity followed by 73% (40/55) for *R. solani*, 55% (24/44) for *P. capsici*, 42% (8/19) of *P. aphanidermatum*, and 53% (33/62) for powdery mildew (of those that significantly suppressed the disease in the leaf disk bioassay at *p* < 0.05). Interestingly, 83% (86/104 RNase positive strains) belonged to *Bacillus* (76/104) and *Paenibacillus* (10/104). In previous studies, *Bacilli* have been shown to produce RNAses, for example, *Bacillus amyloliquefaciens* has been shown to produce barnase, a ribonuclease and antibiotic protein (Khan et al., 2016). Noteworthy, the antifungal activity of some pathogenesis-related (PR) proteins secreted by host plants has been associated with their ribonuclease activity, including the PR-4 and PR-10 families (Filipenko et al., 2013; Miranda et al., 2017; Moosa et al., 2017). The associated mechanism(s) of action of these ribonucleases is not clear but may include direct antagonism (by penetrating fungal cells and degrading native mRNA) or indirect antagonism (by inducing host programmed cell death) (Filipenko et al., 2013; Miranda et al., 2017; Moosa et al., 2017). Further experiments will be required to determine whether the extracellular RNase activity of any of the endophytes plays a role in their phytopathogen suppression, and if so, their mechanism of action.

### Potential Role of Lytic Enzymes in Phytopathogen Suppression

In our previous study (Khalaf and Raizada, 2016), we reported that extracellular lytic enzyme activities, including cellulase, pectinase and protease function, were displayed by numerous cucurbit seed-associated bacterial endophytes. Our previous observation may be relevant here, as several biocontrol agents have been shown to exert their antagonistic activity through secretion of lytic enzymes that protect the plant either directly or indirectly. The direct mechanism has been shown to involve breaking down of essential complex polymers within the pathogen such as chitin, protein, cellulose and DNA (Pliego et al., 2011). By contrast, the lysis products (e.g., chitin fragments) can be indirectly employed in plant protection by eliciting host defence responses (Heydari and Pessarakli, 2010; Pliego et al., 2011; Duran-Flores and Heil, 2016). Further experiments will be needed to verify this hypothesis.

### Study Limitations, Future Experiments and Biocontrol Applications

Surprisingly, we noticed adverse effects from some endophytic bacteria in the cucumber leaf disk bioassay (**Supplementary Table S2B**), suggesting that they were being recognized as pathogens or added to plant stress, perhaps due to their high titre in combination with plant wounding. Some endophytes have been shown to be helpful to specific hosts but pathogenic to others (Brader et al., 2017). Alternatively, some of the observed variation between the endophytes may have been due to differences in their growth phase. In a study by Romero et al. (2004), bacterial cells in stationary phase showed relatively greater inhibition than log phase cells with respect to growth of the obligate pathogen *Podosphaera fuliginea* (Romero et al., 2004). With respect to the strain biosafety, promising endophytes must be subject to further study to evaluate their impacts on human health and the surrounding environment. Potential risks could be identified through the rules and regulations of the safety assessment charter laid down by the American Biological Safety Association (ABSA) (Bharti et al., 2017).

Our study represents only the first step toward the development of novel biocontrol inoculants. Apart from powdery mildew, the study was limited to *in vitro* screening, and likely revealed endophytes that exhibit direct antagonism, along with traits such as VOCs pertinent to the induction of the host defense response. Other implicated biocontrol mechanisms such as pathogen/endophyte-plant interactions and competition with natural microflora will require *in planta* assays (Eljounaidi et al., 2016). More generally, future biological control applications of promising antagonists will require replicated field and greenhouse trials, which are forthcoming.

The focus of this study was bacterial, not fungal, endophytes as potential biocontrol agents, because vegetable growers spray with fungicides, and hence bacterial biocontrol agents can be integrated with chemical approaches. Furthermore, bacteria are easily handled and rapidly grow to permit inoculant preparation (Whipps, 2001). Although very limited, a small number of successful trials of seed bacterial endophytes have been reported for the management of fungal phytopathogens (Mukhopadhyay et al., 1996; Loper and Henkels, 1999; Cottyn et al., 2001; Ruiza et al., 2011; Gagne-Bourgue et al., 2013; Truyens et al., 2015), suggesting that the microbes identified in this study may have future biocontrol potential. Optimistically with the advent of next generation sequencing technology, omics tools could help in performing studies of comparative genomics between *de novo* inoculants and their biocontrol cousins of the same genus. These studies may facilitate the selection of promising candidates for further *in vivo* testing based on their genomes encoding biocontrol genes (Douriet-Gámez et al., 2017).

### Study Implications

Cucurbit fruits and their seeds have been widely grown by humans for thousands of years, able to grow in diverse climates where fungi and oomycetes can thrive. As with all plants, cucurbit seeds germinate in the soil. Here we demonstrate that the majority of seed associated endophytes of cultivated cucurbits have the potential to suppress fungal and oomycete pathogens that affect early seed germination as well as a later foliar disease. Furthermore, we demonstrate that the majority of these antagonistic microbes are *Bacillus* and *Paenabacillus* strains. *Bacilli* are widely used as commercial biocontrol agents of vegetable crops, and it is interesting to speculate whether these biocontrol agents are mimicking the ecological niche established by their endophytic cousins. The endophytes characterized in this study may assist with the implementation of alternative pest management strategies to address growing concerns for safe food production globally.

## Materials and Methods

### Source of Tested Endophytic Bacteria

One hundred and sixty nine endophytic bacteria were previously cultured from seeds of diverse cucurbits and classified based on their 16S rRNA sequences (**Supplementary Table S1**) (Khalaf and Raizada, 2016).

### *In Vitro* Testing of Functional Traits That Promote Host Resistance

#### Acetoin and Diacetyl Production

The method was adapted from previous protocols (Phalip et al., 1994; Johnston-Monje and Raizada, 2011). Autoclaved lysogeny broth (LB) medium (composed of 10 g NaCl, 5 g yeast extract, 10 g tryptone, per liter) was amended with filter sterilized 0.5% glucose and distributed as 1 ml aliquots in 96 deep well plates (#07-200-700, Fisher). The plates were inoculated with overnight grown bacterial endophytes using a flame-sterilized 96-pin replicator, sealed with a breathable membrane and incubated in a shaking incubator (150 rpm at 28°C) for 5 days. On the fifth day, 100 μl of each bacterial culture was transferred to a 96 well white fluorometer plate using a multichannel pipet. Subsequently, 100 μl/well of freshly prepared Barritt’s Reagents A and B was added, and then left to stand for 15 min. The development of red/pink color was scored as a positive result compared to the copper color of the reagent (negative control). For confirmation, the test was performed in triplicate. Barritt’s Reagents A and B were prepared in a proportion of 3:1 (v/v) by mixing 3 parts of 0.5% (w/v) creatine solution with 1 part of freshly prepared 7.5% (w/v) α-naphthol in 2.5 N sodium hydroxide.

#### Ribonuclease Activity

In 10 ml of 0.1 M Na_2_HPO_4_ (pH 8), 1.5 g of torula yeast RNA (#R6625, Sigma–Aldrich) was dissolved, filter sterilized and added to 250 ml of autoclaved LB agar media (Hole et al., 2004). Bacterial endophytes were inoculated onto agar plates using a flame sterilized pin replicator, and incubated at 28°C. After 3 days, 70% perchloric acid was flooded over the plates for 15 min. A clear halo around a colony was scored as a positive result. The test was performed in triplicate.

### *In Vitro* Anti-microbial Screening

#### Source of Plant Pathogens

*The Rhizoctonia solani* strain was isolated from sugar beet cultivated in Ontario, Canada, and identified phenotypically at the University of Guelph (Ridgetown campus) and by Dr. Linda Hanson (United States Department of Agriculture, USDA) in Michigan, United States. *Phytophthora capsici* was isolated from pepper grown in Simcoe Station, Ontario and identified phenotypically by Dr. Catarina Saude (University of Guelph) with completion of Koch’s postulates. *Pythium aphanidermatum* was obtained from cucumber grown in Quebec, identified and supplied by Prof. Mary Ruth McDonald (University of Guelph). *Fusarium graminearum* was isolated from maize and supplied by Agriculture and Agri-food Canada (AAFC), Guelph, ON, Canada.

#### Agar Disk Diffusion Technique

Using the agar disk diffusion technique (Shehata et al., 2016), the tested soil-borne fungal plant pathogens and oomycetes were grown in YPD broth (#Y1375, Sigma–Aldrich) for 3–5 days (based on the fungus growth rate) at 25°C with gentle shaking. Pre-melted cooled PDA media was inoculated with the mycelia (25 ml/150 ml PDA for *R. solani*, 1 ml/50 ml PDA for *P. capsici* and *F. graminearum*, and 1 ml/100ml PDA for *P. aphanidermatum*) and 50 ml were poured into each sterile Petri dish (150 mm × 15 mm), then left to solidify. Using sterile glass tubes, 11 mm diameter cups (wells) were made in the inoculated agar plates. Cups were loaded with 100 μl of overnight LB culture of each bacterial endophyte with adjusted OD_600_ 0.4–0.6. The fungicide Nystatin (N 581, PhytoTechnology Laboratories, United States) was dissolved in dimethyl sulfoxide (DMSO) and used as a positive control (≈202 U/100 μl for *R. solani*, 454 U/100 μl for *F. graminearum* and 1817 U/100 μl for *P. capsici*), and DMSO was used as the negative control. Propamocarb hydrochloride (722 g/L, aqueous solution, UVP:05933765, Bayer CropScience Inc., Canada) was used as a positive control for *P. aphanidermatum* at its commercial concentration, and autoclaved ddH_2_O was used as the negative control. The plates were incubated at 25°C for 3–5 days in darkness. The anti-microbial activity was scored by measuring the diameter of the inhibition zone around each agar cup. The assay was performed in triplicate for each bacterial endophyte.

### *In Planta* Techniques for Screening for Antagonism against Powdery Mildew

#### Plant Variety and Growth Conditions

The National Pickling cucumber variety was purchased from William Dam Seeds Ltd. (Dundas, ON, Canada). Seeds were germinated for 4–6 days, then transplanted and grown in a growth room at 25/18°C for 16/8 h (day/night), respectively. The humidity was maintained at 75% during the experiment, and the light intensity (cool white fluorescent bulbs) was measured (150–220 μmol m^-2^ sec^-1^) at the top of the plants using a photon flux meter in the photosynthetically active radiation (PAR) range of 400–700 nm (model BQM-01, Apogee Instruments Inc., Logan, UT, United States). For the leaf biocontrol assays, 30–35 days old plants (after sowing) were used.

#### Source and Maintenance of the Pathogen

As *Podosphaera fuliginea* is an obligate pathogen, cucumber leaves infected with powdery mildew were obtained from a commercial greenhouse (Leamington, ON, Canada) in collaboration with the Ontario Ministry of Agriculture, Food and Rural Affairs. The causative pathogen was maintained on leaves of healthy cucumber plants (National Pickling cucumber variety) by tapping the infected leaves over the healthy ones to mechanically transfer the fungal spores. The leaves were then gently misted with water droplets, and the plants were covered with plastic bags for 24 h to raise the humidity. The bags were then removed and the plants were maintained in a growth chamber at 24/18°C for 16/8 h (day/night). The humidity was adjusted to 60% during the day and 80% at night, and the light intensity was as noted above. The early signs of disease appeared on the infected leaves within 1 week.

#### Molecular Identification of the Powdery Mildew Causative Pathogen

A heavily infected leaf was selected and the conidia were collected by spraying with autoclaved 0.01% Triton X-100 in ddH_2_O. The spore suspension was centrifuged and washed using the same solution at 10,000 rpm for 10 min. The pelleted spores were ground under liquid nitrogen in a mortar and pestle (Chen et al., 2008; Glawe, 2008). Fungal DNA was isolated using a DNeasy^®^Plant Mini kit (#69106, Qiagen, United States) and quantified using a Nanodrop (Thermo Scientific, United States). PCR was performed using primer pair ITS4 (forward, 5′-TCCTCCGCTTATTGATATGC-3′) and ITS1F (reverse, 5′-CTTGGTCATTTAGAGGAAGTAA-3′) (Amend et al., 2010). Approximately 50 ng of total DNA was added to a PCR mixture containing 4 μl Standard Taq Buffer (M791B, Promega), 0.4 μl of 25 mM dNTP mix, 0.5 μl of each primer (10 mM working stock), 0.6 μl of 50 mM MgCl_2_, 0.2 μl of Standard Taq (New England Biolabs) and H_2_O to a final volume of 20 μl. A PCR reaction was undertaken using the following conditions: an initial denaturation at 95°C for 10 min, then 34 cycles (95°C for 1 min, 51°C for 1 min, 72°C for 1 min) and a final extension at 72°C for 7 min. Amplicons (600 bp) were gel purified, sequenced and then searched against fungal ITS deposits within RDP (Ribosomal Database Project) and also BLASTN searched against NCBI using the default parameters (**Supplementary Table S2E**).

#### Leaf Disk Bioassay

Adapting a previous protocol (Sun et al., 2013), the leaves from the 2nd to 5th node of cucumber plants were detached and 0.5 inch-diameter leaf-disks were created by using a paper puncher. Fifteen leaf-disks were used for each endophytic bacterium (3 Petri dishes, 5 disks per dish) by soaking them in a bacterial suspension of LB broth amended with filter sterilized 0.01% Tween 20 and adjusted to OD_600_ ∼0.3–0.5 for 1 min with gentle shaking. Then five disks were transferred into each Petri dish (6 cm diameter) using flame sterilized forceps where they were placed on a damp filter paper (5.5 cm filter paper Whatman No.1) wetted with 2 ml of nutrient solution optimized for cucumbers [per 1 L of tap water: 950 mg Ca(NO_3_)_2_].4H_2_O; 810 mg KNO_3_; 500 mg MgSO_4_; 7H_2_O; 155 mg NH_4_H_2_PO_4_; 3 mg H_3_BO_3_; 2 mg; ZnSO4.7H_2_O; 0.05 mg CuSO_4_.5H_2_O; 0.02 mg NaMoO_4_; and 25 mg NaFeEDTA (Pramanik et al., 2000). The leaf disks were placed with their adaxial side up. The Petri dishes were incubated in darkness at 30°C for 24 h. After 24 h, the conidial suspension was prepared by adapting a previous protocol (Kim et al., 2007): 10 μl of the freshly prepared conidial suspension (adjusted to 1 × 10^5^ CFU/ml, in autoclaved ddH_2_O supplemented with 0.01% Triton X-100) were placed on the center of each leaf disk as a pathogen inoculum. The Petri dishes were then sealed with breathable tape and incubated in a growth chamber using the same conditions used for pathogen maintenance (above). Prothioconazole fungicide (used as 24 μg/ml concentration and prepared from 480 g/L stock solution) and a commercial biocontrol agent (*Bacillus subtilis* strain QST 713, formulated in a 1 mg/ml suspension of water dispersible granules) were used as positive controls.

LB broth supplemented with filter sterilized 0.01% Tween 20 was used as a negative control. Visual assessment of the disease severity was performed using a 0–7 scale based on the development of infected lesions and chlorosis, by cross-referencing to selected diseased leaf photos corresponding to different levels of disease severity (**Figure 4F**). The disease index and preventive effect were calculated using the following equations:

$$Disease\,\;index(\%)=\frac{\Sigma \,\text{ Number of diseased leaves at each disease scoring scale value }\times \text{ the corresponding disease scoring scale value}}{\text{Total number of investigated leaves }\times \text{ the highest disease scoring scale value}}\times 100$$

$$\text{Preventive effect }(\%)=\frac{\text{Disease index of negative control}-\text{disease index of treatment}}{\text{Disease index of negative control}}\times 100$$

#### Detached Whole Leaf Bioassay

Adapting a previous protocol (Sun et al., 2013), whole cucumber leaves from the 2nd – 5th node were selected, and each leaf was placed on a damp filter paper wetted with the same nutrient solution used in leaf disk bioassay and placed in a 150 mm diameter Petri dish. The petiole of each leaf was wrapped in an autoclaved, wetted (with cucumber nutrient solution) cotton puff. Overnight endophytic bacterial cultures were harvested and re-suspended in LB broth supplemented with 0.01% Tween 20 and adjusted to an OD_600_ ∼0.3–0.5. Each leaf was sprayed with a ∼5 ml bacterial suspension using a hand sprayer. Petri dishes were incubated at 30°C in darkness. After 24 h, the leaves were sprayed with ∼0.5 ml of conidial suspension (1 × 10^5^ CFU/ml) using a hand sprayer. The Petri dishes were sealed with breathable tape and incubated in a growth chamber for 7 days (under the same conditions used for pathogen maintenance). The same positive and negative controls were used. For each treatment including controls there were five leaves representing five replicates. The disease severity was performed visually using the same 0–7 scale as noted above. The disease index and preventive effects were calculated using the same equations utilized in the leaf disk bioassay.

### Statistical Analysis

Data of leaf disk bioassay were analyzed using GraphPad Prism 7. One-way analysis of variance (ANOVA) was applied, and mean values of the treatments were compared to controls using Dunnett’s multiple comparison test. Data from the detached whole leaf bioassay were analyzed using *t*-tests of visual disease scores (one-tailed and unequal variance).

## Author Contributions

MR designed the research and helped to write the manuscript. EK performed all the lab work, data analysis and wrote the manuscript. All authors read and approved the final manuscript.

## Conflict of Interest Statement

The authors declare that the research was conducted in the absence of any commercial or financial relationships that could be construed as a potential conflict of interest.

## Supplementary Material

FIGURE S1 Selected powdery mildew disease leaf photos corresponding to different levels of disease severity used as references to guide visual disease assessments of the detached whole leaf bioassay. **(A–E)** Shown are representative photos of tested cucumber leaves: **(A)** chemical fungicide positive control (Prothioconazole), **(B)** commercial biocontrol agent positive control (*Bacillus subtilis* strain QST 713), **(C)** example of a typical promising endophytic bacteria; *Pantoea* EKM103V, **(D)** negative control (LB broth amended with filter sterilized 0.01% Tween 20), **(E)** example of an inoculant *Bacillus*_EKM502B with adverse effect. R (1–5) denotes replicate number.

TABLE S1 Detailed information of *in vitro* phenotypic traits and antimicrobial activity of cucurbit seed bacterial endophytes tested in this study.

TABLE S2 Detailed information of the effect of the cucurbit seed bacterial endophytes on powdery mildew disease using the leaf disk bioassay and detached whole leaf bioassay.

TABLE S2A Summary of the leaf disk bioassay results for all endophytes tested against powdery mildew, with their corresponding Genbank accession numbers, organized by bacterial phylum, genus and original host seed source. A greater Disease Index indicates increased disease symptoms, whereas a greater Preventative Effect denotes improved control of the disease.

TABLE S2B Raw data and calculations for Disease Index, corresponding to Supplementary Table S2A (leaf disk bioassay).

TABLE S2C Raw data and calculations for Preventative Effect, corresponding to S2A (leaf disk bioassay).

TABLE S2D Detailed information of the detached whole leaf bioassay results for all endophytes tested against powdery mildew, with their corresponding Genbank accession numbers, organized by bacterial phylum, genus and original host seed source. A greater Disease Index indicates increased disease symptoms, whereas a greater Preventative Effect denotes improved control of the disease.

TABLE S2E Molecular identification (ITS rDNA) of the powdery mildew isolate used in this study.

TABLE S3 Complete list of endophytic strains displaying a positive preventative effect (PE) against powdery mildew in the leaf disk bioassay, organized by bacterial phylum, genus and original seed source. Statistical analysis (Dunnett’s Multiple Comparison Test) of the disease index percentages (DI%) per treatment (*n* = 3) compared to the negative control is represented as calculated p values. A greater Disease Index indicates increased disease symptoms, whereas a greater Preventative Effect denotes improved control of the disease. The circumflex accent (ˆ) denotes that fresh fruits were used as the seed source. NS, non-significant, ^∗^*p* < 0.05, ^∗∗^*p* < 0.01, ^∗∗∗^*p* < 0.001, ^∗∗∗∗^*p* < 0.0001.

TABLE S4 Raw data and graphical representation of calculated means of the disease index percentage (DI%) displayed by endophytic bacteria that exhibited significant suppression of powdery mildew (*p* < 0.05) in the leaf disk bioassay along with their corresponding standard deviation and standard error of mean (SEM). Error bars show the SEM.

## References

[B1] Agrios G. N. (ed.) (2005). *Plant Pathology*, 5th Edn San Diego, CA: Academic Press.

[B2] Akbar A. Ali I. Anal A. K. (2016). Industrial perspectives of lactic acid bacteria for biopreservation and food safety. *J. Anim. Plant Sci.* 26 938–948.

[B3] Amend A. S. Seifert K. A. Samson R. Bruns T. D. (2010). Indoor fungal composition is geographically patterned and more diverse in temperate zones than in the tropics. *Proc. Natl. Acad. Sci. U.S.A.* 107 13748–13753. 10.1073/pnas.1000454107 20616017 PMC2922287

[B4] Asari S. Matzén S. Petersen M. A. Bejai S. (2016). Multiple effects of *Bacillus amyloliquefaciens* volatile compounds: plant growth promotion and growth inhibition of phytopathogens. *FEMS Microbiol. Ecol.* 92:fiw070. 10.1093/femsec/fiw070 27053756

[B5] Bacon C. W. White J. F. (2016). Functions, mechanisms and regulation of endophytic and epiphytic microbial communities of plants. *Symbiosis* 68 87–98. 10.1007/s13199-015-0350-2

[B6] Berg G. (2009). Plant-microbe interactions promoting plant growth and health: perspectives for controlled use of microorganisms in agriculture. *Appl. Microbiol. Biotechnol.* 84 11–18. 10.1007/s00253-009-2092-7 19568745

[B7] Berg G. Köberl M. Rybakova D. Müller H. Grosch R. Smalla K. (2017). Plant microbial diversity is suggested as the key to future biocontrol and health trends. *FEMS Microbiol. Ecol.* 93:fix050. 10.1093/femsec/fix050 28430944

[B8] Bharti N. Sharma S. K. Saini S. Verma A. Nimonkar Y. Prakash O. (2017). “Microbial plant probiotics: problems in application and formulation,” in *Probiotics and Plant Health*, eds Kumar V. Kumar M. Sharma S. Prasad R. (Singapore: Springer Nature). 10.1007/978-981-10-3473-2

[B9] Bitas V. Kim H.-S. Bennett J. W. Kang S. (2013). Sniffing on microbes: diverse roles of microbial volatile organic compounds in plant health. *Mol. Plant Microbe Interact.* 26 835–843. 10.1094/MPMI-10-12-0249-CR 23581824

[B10] Borneman J. Skroch P. W. O’Sullivan K. M. Palus J. A. Rumjanek N. G. Jansen J. L. (1996). Molecular microbial diversity of an agricultural soil in Wisconsin. *Appl. Environ. Microbiol.* 62 1935–1943. 8787391 10.1128/aem.62.6.1935-1943.1996 PMC167971

[B11] Bosmans L. De Bruijn I. De Mot R. Rediers H. Lievens B. (2016). Agar composition affects in vitro screening of biocontrol activity of antagonistic microorganisms. *J. Microbiol. Methods* 127 7–9. 10.1016/j.mimet.2016.05.004 27166668

[B12] Brader G. Compant S. Vescio K. Mitter B. Trognitz F. Ma L.-J. (2017). Ecology and genomic insights on plant-pathogenic and -nonpathogenic endophytes. *Annu. Rev. Phytopathol.* 55 61–83. 10.1146/annurev-phyto-080516-035641 28489497

[B13] Braun U. Cook R. T. A. (eds) (2012). *Taxonomic Manual of the Erysiphales.* Utrecht: CBS-KNAW Fungal Biodiversity Centre.

[B14] Böhme M. H. Pinker I. Junge H. (2016). “Effect of organic biostimulators with *Bacillus amyloliquefaciens* ssp. Plantarum (former *Bacillus subtilis*) as the main agent in vegetable cultivation,” in *Bacilli and Agrobiotechnology*, eds Islam M. T. Rahman M. Pandey P. Jha C. K. Aeron A. (Cham: Springer). 10.1007/978-3-319-44409-3

[B15] Chen R. S. Chu C. Cheng C. W. Chen W. Y. Tsay J. G. (2008). Differentiation of two powdery mildews of sunflower (*Helianthus annuus*) by a PCR-mediated method based on ITS sequences. *Eur. J. Plant Pathol.* 121 1–8. 10.1007/s10658-007-9234-5

[B16] Choudhary D. K. Johri B. N. (2009). Interactions of *Bacillus* spp. and plants - With special reference to induced systemic resistance (ISR). *Microbiol. Res.* 164 493–513. 10.1016/j.micres.2008.08.007 18845426

[B17] Chung J. Song G. C. Ryu C.-M. (2016). Sweet scents from good bacteria: case studies on bacterial volatile compounds for plant growth and immunity. *Plant Mol. Biol.* 90 677–687. 10.1007/s11103-015-0344-8 26177913

[B18] Cohen R. (1993). A leaf disk assay for detection of resistance of melons to *Sphaerotheca fuliginea* race 1. *Plant Dis.* 77 513–517.

[B19] Cottyn B. Regalado E. Lanoot B. De Cleene M. Mew T. W. Swings J. (2001). Bacterial populations associated with rice seed in the tropical environment. *Phytopathology* 91 282–292. 10.1094/PHYTO.2001.91.3.282 18943348

[B20] Dean R. Van Kan J. A. L. Pretorus Z. A. Hammond-Kosack K. E. Di Pietro A. Spanu P. D. (2012). The Top 10 fungal pathogens in molecular plant pathology. *Mol. Plant Pathol.* 13 414–430. 10.1111/j.1364-3703.2011.00783.x 22471698 PMC6638784

[B21] Díaz Herrera S. Grossi C. Zawoznik M. Groppa M. D. (2016). Wheat seeds promoters harbour bacterial endophytes with potential as plant growth of biocontrol agents *Fusarium graminearum*. *Microbiol. Res.* 186–187, 37–43. 10.1016/j.micres.2016.03.002 27242141

[B22] Douriet-Gámez N. R. Maldonado-Mendoza I. E. Ibarra-Laclette E. Blom J. Calderón-Vázquez C. L. (2017). Genomic analysis of *Bacillus* sp. Strain B25 a biocontrol agent of maize pathogen *Fusarium verticillioides*. *Curr. Microbiol.* 10.1007/s00284-017-1372-1 [Epub ahead of print] 29051980

[B23] Duran-Flores D. Heil M. (2016). Sources of specificity in plant damaged-self recognition. *Curr. Opin. Plant Biol.* 32 77–87. 10.1016/j.pbi.2016.06.019 27421107

[B24] Eljounaidi K. Lee S. K. Bae H. (2016). Bacterial endophytes as potential biocontrol agents of vascular wilt diseases – Review and future prospects. *Biol. Control* 103 62–68. 10.1016/j.biocontrol.2016.07.013

[B25] Emmert E. A. Handelsman J. (1999). Biocontrol of plant disease: a (gram-) positive perspective. *FEMS Microbiol. Lett.* 171 1–9. 10.1111/j.1574-6968.1999.tb13405.x 9987836

[B26] Filipenko E. A. Kochetov A. V. Kanayama Y. Malinovsky V. I. Shumny V. K. (2013). PR-proteins with ribonuclease activity and plant resistance against pathogenic fungi. *Russ. J. Genet. Appl. Res.* 3 474–480. 10.1134/S2079059713060026

[B27] Fürnkranz M. Adam E. Müller H. Grube M. Huss H. Winkler J. (2012). Promotion of growth, health and stress tolerance of Styrian oil pumpkins by bacterial endophytes. *Eur. J. Plant Pathol.* 134 509–519. 10.1007/s10658-012-0033-2

[B28] Gagne-Bourgue F. Aliferis K. A. Seguin P. Rani M. Samson R. Jabaji S. (2013). Isolation and characterization of indigenous endophytic bacteria associated with leaves of switchgrass (*Panicum virgatum* L.) cultivars. *J. Appl. Microbiol.* 114 836–853. 10.1111/jam.12088 23190162

[B29] Gajbhiye M. H. Kapadnis B. P. (2016). Antifungal-activity-producing lactic acid bacteria as biocontrol agents in plants. *Biocontrol Sci. Technol.* 26 1451–1470. 10.1080/09583157.2016.1213793

[B30] Garbeva P. Van Veen J. A. Van Elsas J. D. (2003). Predominant *Bacillus* spp. in agricultural soil under different management regimes detected via PCR-DGGE. *Microb. Ecol.* 45 302–316. 10.1007/s00248-002-2034-8 12632212

[B31] García-Gutiérrez L. Romero D. Zeriouh H. Cazorla F. M. Torés J. A. de Vicente A. (2012). Isolation and selection of plant growth-promoting rhizobacteria as inducers of systemic resistance in melon. *Plant Soil* 358 201–212. 10.1007/s11104-012-1173-z

[B32] García-Gutiérrez L. Zeriouh H. Romero D. Cubero J. de Vicente A. Pérez-García A. (2013). The antagonistic strain *Bacillus subtilis* UMAF6639 also confers protection to melon plants against cucurbit powdery mildew by activation of jasmonate- and salicylic acid-dependent defence responses. *Microb. Biotechnol.* 6 264–274. 10.1111/1751-7915.12028 23302493 PMC3815921

[B33] Glassner H. Zchori-Fein E. Compant S. Sessitsch A. Katzir N. Portnoy V. (2015). Characterization of endophytic bacteria from cucurbit fruits with potential benefits to agriculture in melons (*Cucumis melo* L.). *FEMS Microbiol. Ecol.* 91 1–13. 10.1093/femsec/fiv074 26183916

[B34] Glawe D. A. (2008). The powdery mildews: a review of the world’s most familiar (yet poorly known) plant pathogens. *Annu. Rev. Phytopathol.* 46 27–51. 10.1146/annurev.phyto.46.081407.104740 18680422

[B35] Hardoim P. R. van Overbeek L. S. Berg G. Pirttilä A. M. Compant S. Campisano A. (2015). The hidden world within plants: ecological and evolutionary considerations for defining functioning of microbial endophytes. *Microbiol. Mol. Biol. Rev.* 79 293–320. 10.1128/MMBR.00050-14 26136581 PMC4488371

[B36] Hausbeck M. K. Lamour K. H. (2004). *Phytophthora capsici* on vegetable crops: research progress and management challenges. *Plant Dis.* 88 1292–1303. 10.1094/PDIS.2004.88.12.1292 30795189

[B37] Heneidak S. Khalik K. A. (2015). Seed coat diversity in some tribes of Cucurbitaceae: implications for taxonomy and species identification. *Acta Bot. Bras.* 29 129–142. 10.1590/0102-33062014abb3705

[B38] Heydari A. Pessarakli M. (2010). A review on biological control of fungal plant pathogens using microbial antagonists. *J. Biol. Sci.* 10 273–290.

[B39] Hole R. C. Singhal R. S. Melo J. S. D’Souza S. F. (2004). A rapid plate screening technique for extracellular ribonuclease producing strains. *BARC Newsl.* 249 91–97.

[B40] Huang Y. Kuang Z. Wang W. Cao L. (2016). Exploring potential bacterial and fungal biocontrol agents transmitted from seeds to sprouts of wheat. *Biol. Control* 98 27–33. 10.1016/j.biocontrol.2016.02.013

[B41] Jeffrey C. (1980). A review of the Cucurbitaceae. *Biol. J. Linn. Soc.* 81 233–247. 10.1111/j.1095-8339.1980.tb01676.x

[B42] Johnson K. B. Stockwell V. O. (1998). Management of fire blight: a case study in microbial ecology. *Annu. Rev. Phytopathol.* 36 227–248. 15012499 10.1146/annurev.phyto.36.1.227

[B43] Johnston-Monje D. Lundberg D. S. Lazarovits G. Reis V. M. Raizada M. N. (2016). Bacterial populations in juvenile maize rhizospheres originate from both seed and soil. *Plant Soil* 405 337–355. 10.1007/s11104-016-2826-0

[B44] Johnston-Monje D. Raizada M. N. (2011). Conservation and diversity of seed associated endophytes in *Zea* across boundaries of evolution, ethnography and ecology. *PLOS ONE* 6:e20396. 10.1371/journal.pone.0020396 21673982 PMC3108599

[B45] Justyna N. Magdalena S. Urszula M. (2017). *Trichoderma atroviride* enhances phenolic synthesis and cucumber protection against *Rhizoctonia solani*. *Plant Prot. Sci.* 53 2007–2013. 10.17221/126/2016-PPS

[B46] Kandel S. L. Firrincieli A. Joubert P. M. Okubara P. Natalie D. Mcgeorge K. M. (2017). An in vitro study of bio-control and plant growth promotion potential of Salicaceae endophytes. *Front. Microbiol.* 8:386. 10.3389/fmicb.2017.00386 28348550 PMC5347143

[B47] Karimi E. Safaie N. Shams-Baksh M. Mahmoudi B. (2016). *Bacillus amyloliquefaciens* SB14 from rhizosphere alleviates *Rhizoctonia* damping-off disease on sugar beet. *Microbiol. Res.* 192 221–230. 10.1016/j.micres.2016.06.011 27664740

[B48] Kates H. R. Soltis P. S. Soltis D. E. (2017). Evolutionary and domestication history of *Cucurbita* (pumpkin and squash) species inferred from 44 nuclear loci. *Mol. Phylogenet. Evol.* 111 98–109. 10.1016/j.ympev.2017.03.002 28288944

[B49] Khalaf E. M. Raizada M. N. (2016). Taxonomic and functional diversity of cultured seed associated microbes of the cucurbit family. *BMC Microbiol.* 16:131. 10.1186/s12866-016-0743-2 27349509 PMC4924336

[B50] Khan A. Doshi H. V. Thakur M. C. (2016). “A profilic siderophore producer,” in *Bacilli and Agrobiotechnology*, eds Islam M. T. Rahman M. Pandey P. Jha C. K. Aeron A. (Cham: Springer). 10.1007/978-3-319-44409-3

[B51] Kim J. J. Goettel M. S. Gillespie D. R. (2007). Potential of *Lecanicillium* species for dual microbial control of aphids and the cucumber powdery mildew fungus, *Sphaerotheca fuliginea*. *Biol. Control* 40 327–332. 10.1016/j.biocontrol.2006.12.002

[B52] Lamichhane J. R. Dürr C. Schwanck A. A. Robin M. H. Sarthou J. P. Cellier V. (2017). Integrated management of damping-off diseases. A review. *Agron. Sustain. Dev.* 37:10. 10.1007/s13593-017-0417-y 21355993

[B53] Larousse M. Galiana E. (2017). Microbial partnerships of pathogenic oomycetes. *PLOS Pathog.* 13:e1006028. 10.1371/journal.ppat.1006028 28125714 PMC5268404

[B54] Lebeda A. Køístková E. Sedláková B. McCreight J. D. Coffey M. D. (2016). Cucurbit powdery mildews: methodology for objective determination and denomination of races. *Eur. J. Plant Pathol.* 144 399–410. 10.1007/s10658-015-0776-7

[B55] Lee B. Farag M. A. Park H. B. Kloepper J. W. Lee S. H. Ryu C. M. (2012). Induced resistance by a long-chain bacterial volatile: elicitation of plant systemic defense by a C13 volatile produced by *Paenibacillus polymyxa*. *PLOS ONE* 7:e48744. 10.1371/journal.pone.0048744 23209558 PMC3509098

[B56] Liu Y. Bai F. Li N. Wang W. Cheng C. (2017). Identification of endophytic bacterial strain RSE1 from seeds of super hybrid rice Shenliangyou 5814 (*Oryza sativa* L.,) and evaluation of its antagonistic activity. *Plant Growth Regul.* 82 403–408. 10.1007/s10725-017-0265-4

[B57] Loper J. E. Henkels M. D. (1999). Utilization of heterologous siderophores enhances levels of iron available to *Pseudomonas putida* in the rhizosphere. *Appl. Environ. Microbiol.* 65 5357–5363. 10583989 10.1128/aem.65.12.5357-5363.1999 PMC91729

[B58] Mahaffee W. F. Kloepper J. W. (1997). Temporal changes in the bacterial communities of soil, rhizosphere, and endorhiza associated with field-grown cucumber (*Cucumis sativus* L.). *Microb. Ecol.* 34 210–223. 10.1007/s002489900050 9337416

[B59] Mandal V. Sen S. K. Mandal N. C. (2007). Detection, isolation and partial characterization of antifungal compound(s) produced by *Pediococcus acidilactici* LAB 5. *Nat. Prod. Commun.* 2 671–674.

[B60] McSpadden Gardener B. B. (2004). Ecology of *Bacillus* and *Paenibacillus* spp. in agricultural systems. *Phytopathology* 94 1252–1258. 10.1094/PHYTO.2004.94.11.1252 18944463

[B61] McSpadden Gardener B. B. Fravel D. R. (2002). Biological control of plant pathogens: research, commercialization, and application in the USA. *Plant Health Prog.* 10.1094/PHP-2002-0510-01-RV

[B62] Miranda V. de J. Porto W. F. Nolasco D. O. Claudia A. Araujo G. (2017). Comparative transcriptomic analysis indicates genes associated with local and systemic resistance to *Colletotrichum graminicola* in maize. *Sci. Rep.* 7:2483. 10.1038/s41598-017-02298-8 28559543 PMC5449407

[B63] Mitter B. Pfaffenbichler N. Sessitsch A. (2016). Plant-microbe partnerships in 2020. *Microb. Biotechnol.* 9 635–640. 10.1111/1751-7915.12382 27418200 PMC4993182

[B64] Moosa A. Farzand A. Sahi S. T. Khan S. A. (2017). Transgenic expression of antifungal pathogenesis-related proteins against phytopathogenic fungi – 15 years of success. *Isr. J. Plant Sci.* 10.1080/07929978.2017.1288407

[B65] Mousa W. K. Shearer C. Limay-Rios V. Ettinger C. L. Eisen J. A. Raizada M. N. (2016). Root-hair endophyte stacking in finger millet creates a physicochemical barrier to trap the fungal pathogen *Fusarium graminearum*. *Nat. Microbiol.* 1:16167. 10.1038/nmicrobiol.2016.167 27669453

[B66] Mousa W. K. Shearer C. R. Limay-Rios V. Zhou T. Raizada M. N. (2015). Bacterial endophytes from wild maize suppress Fusarium graminearum in modern maize and inhibit mycotoxin accumulation. *Front. Plant Sci.* 6:805. 10.3389/fpls.2015.00805 26500660 PMC4593954

[B67] Mukhopadhyay K. Garrison N. K. Hinton D. M. Bacon C. W. Khush G. S. Peck H. D. (1996). Identification and characterization of bacterial endophytes of rice. *Mycopathologia* 134 151–159. 10.1007/BF00436723 20882464

[B68] Muraschi T. F. Friend M. Bolles D. (1965). Erwinia-like microorganisms isolated from animal and human hosts. *Appl. Microbiol.* 13 128–131. 14325868 10.1128/am.13.2.128-131.1965 PMC1058210

[B69] Nadarasah G. Stavrinides J. (2014). Quantitative evaluation of the host-colonizing capabilities of the enteric bacterium *Pantoea* using plant and insect hosts. *Microbiology* 160 602–615. 10.1099/mic.0.073452-0 24430494

[B70] Nagórska K. Bikowski M. Obuchowski M. (2007). Multicellular behaviour and production of a wide variety of toxic substances support usage of *Bacillus subtilis* as a powerful biocontrol agent. *Acta Biochim. Pol.* 54 495–508. 17882321

[B71] Nelson E. B. (2017). The seed microbiome: origins, interactions, and impacts. *Plant Soil* 10.1007/s11104-017-3289-7

[B72] Ongena M. Jacques P. (2008). *Bacillus* lipopeptides: versatile weapons for plant disease biocontrol. *Trends Microbiol.* 16 115–125. 10.1016/j.tim.2007.12.009 18289856

[B73] Paris H. S. (2016a). Germplasm enhancement of *Cucurbita pepo* (pumpkin, squash, gourd: Cucurbitaceae): progress and challenges. *Euphytica* 208 415–438. 10.1007/s10681-015-1605-y

[B74] Paris H. S. (2016b). Overview of the origins and history of the five major cucurbit crops: issues for ancient DNA analysis of archaeological specimens. *Veg. Hist. Archaeobot.* 25 1–10. 10.1007/s00334-016-0555-1

[B75] Pessarakli M. (ed.) (2016). *Handbook of Cucurbits: Growth, Cultural Practices, and Physiology.* Boca Raton, FL: CRC Press.

[B76] Pető Á. Kenéz Á. Lisztes-Szabó Z. Sramkó G. Laczkó L. Molnár M. (2016). The first archaeobotanical evidence of *Lagenaria siceraria* from the territory of Hungary: histology, phytoliths and (a)DNA. *Veg. Hist. Archaeobot.* 26 125–142. 10.1007/s00334-016-0566-y

[B77] Phalip V. Schmitt P. Divies C. (1994). A method for screening diacetyl and acetoin-producing bacteria on agar plates. *J. Basic Microbiol.* 34 277–280.

[B78] Pliego C. Ramos C. de Vicente A. Cazorla F. M. (2011). Screening for candidate bacterial biocontrol agents against soilborne fungal plant pathogens. *Plant Soil* 340 505–520. 10.1007/s11104-010-0615-8

[B79] Porto M. C. W. Kuniyoshi T. M. Azevedo P. O. Vitolo M. de Souza Oliveira R. P. (2017). *Pediococcus* spp.: an important genus of lactic acid bacteria and pediocin producers. *Biotechnol. Adv.* 35 361–374. 10.1016/j.biotechadv.2017.03.004 28284993

[B80] Pramanik M. H. R. Nagai M. Asao T. Matsui Y. (2000). Effects of temperature and photoperiod on phytotoxic root exudates of cucumber (*Cucumis sativus*) in hydroponic culture. *J. Chem. Ecol.* 26 1953–1967.

[B81] Punja Z. K. Yip R. (2003). Biological control of damping-off and root rot caused by *Pythium aphanidermatum* on greenhouse cucumbers. *Can. J. Plant Pathol.* 25 411–417. 10.1080/07060660309507098 19120624

[B82] Pusey P. L. (2002). Biological control agents for fire blight of apple compared under conditions limiting natural dispersal. *Plant Dis.* 86 639–644. 10.1094/PDIS.2002.86.6.639 30823238

[B83] Raza W. Ling N. Zhang R. Huang Q. Xu Y. Shen Q. (2017). Success evaluation of the biological control of *Fusarium* wilts of cucumber, banana, and tomato since 2000 and future research strategies. *Crit. Rev. Biotechnol.* 37 202–212. 10.3109/07388551.2015.1130683 26810104

[B84] Renner S. S. Schaefer H. Kocyan A. (2007). Phylogenetics of *Cucumis* (Cucurbitaceae): Cucumber (*C. sativus*) belongs in an Asian/Australian clade far from melon (*C. melo*). *BMC Evol. Biol.* 7:58. 10.1186/1471-2148-7-58 17425784 PMC3225884

[B85] Robinson R. W. Decker-Walters D. S. (1997). *Cucurbits (Crop Production Science in Horticulture)*, 1st Edn Wallingford: CAB international.

[B86] Romero D. Pérez-García A. Rivera M. E. Cazorla F. M. De Vicente A. (2004). Isolation and evaluation of antagonistic bacteria towards the cucurbit powdery mildew fungus *Podosphaera fusca*. *Appl. Microbiol. Biotechnol.* 64 263–269. 10.1007/s00253-003-1439-8 13680203

[B87] Ruiza D. Agaras B. de Werrab P. Wall L. G. Valverde C. (2011). Characterization and screening of plant probiotic traits of bacteria isolated from rice seeds cultivated in Argentina. *J. Microbiol.* 49 902–912. 10.1007/s12275-011-1073-6 22203552

[B88] Rybakova D. Cernava T. Köberl M. Liebminger S. Etemadi M. Berg G. (2016). Endophytes-assisted biocontrol: novel insights in ecology and the mode of action of *Paenibacillus*. *Plant Soil* 405 125–140. 10.1007/s11104-015-2526-1

[B89] Ryu C.-M. Farag M. A. Hu C.-H. Reddy M. S. Kloepper J. W. Paré P. W. (2004). Bacterial volatiles induce systemic resistance in Arabidopsis. *Plant Physiol.* 134 1017–1026. 10.1104/pp.103.026583 14976231 PMC389924

[B90] Ryu C.-M. Farag M. A. Hu C.-H. Reddy M. S. Wei H.-X. Pare P. W. (2003). Bacterial volatiles promote growth in *Arapidopsis*. *Proc. Natl. Acad. Sci. U.S.A.* 100 4927–4932. 12684534 10.1073/pnas.0730845100 PMC153657

[B91] Sandhya V. Shrivastava M. Ali S. Z. Sai Shiva Krishna Prasad V. (2017). Endophytes from maize with plant growth promotion and biocontrol activity under drought stress. *Russ. Agric. Sci.* 43 22–34. 10.3103/S1068367417010165

[B92] Schaefer H. Heibl C. Renner S. S. (2009). Gourds afloat: a dated phylogeny reveals an Asian origin of the gourd family (Cucurbitaceae) and numerous oversea dispersal events. *Proc. R. Soc. B Biol. Sci.* 276 843–851. 10.1098/rspb.2008.1447 19033142 PMC2664369

[B93] Sebastian P. Schaefer H. Telford I. R. H. Renner S. S. (2010). Cucumber (*Cucumis sativus*) and melon (*C. melo*) have numerous wild relatives in Asia and Australia, and the sister species of melon is from Australia. *Proc. Natl. Acad. Sci. U.S.A.* 107 14269–14273. 10.1073/pnas.1005338107 20656934 PMC2922565

[B94] Sellamani M. Kalagatur N. K. Siddaiah C. Mudili V. Krishna K. Natarajan G. (2016). Antifungal and zearalenone inhibitory activity of *Pediococcus pentosaceus* isolated from dairy products on *Fusarium graminearum*. *Front. Microbiol.* 7:890. 10.3389/fmicb.2016.00890 27379035 PMC4904835

[B95] Shehata H. R. Lyons E. M. Jordan K. S. Raizada M. N. (2016). Relevance of in vitro agar based screens to characterize the anti-fungal activities of bacterial endophyte communities. *BMC Microbiol.* 16:7. 10.1186/s12866-016-0623-9 26772737 PMC4715354

[B96] Shi L. Du N. Shu S. Sun J. Li S. Guo S. (2017). *Paenibacillus polymyxa* NSY50 suppresses *Fusarium* wilt in cucumbers by regulating the rhizospheric microbial community. *Sci. Rep.* 7:41234. 10.1038/srep41234 28198807 PMC5304210

[B97] Shishkoff N. (2000). The name of the cucurbit powdery mildew: *Podosphaera* (sect. *Sphaerotheca) xanthii* (Castag.) U. Braun & N. Shish. comb. nov. *Phytopathology* 90 1077–1093.

[B98] Specht K. Siebert R. Hartmann I. Freisinger U. B. Sawicka M. Werner A. (2014). Urban agriculture of the future: an overview of sustainability aspects of food production in and on buildings. *Agric. Hum. Values* 31 33–51. 10.1007/s10460-013-9448-4

[B99] Strange R. N. Scott P. R. (2005). Plant disease: a threat to global food security. *Annu. Rev. Phytopathol.* 43 83–116. 10.1146/annurev.phyto.43.113004.133839 16078878

[B100] Strausbaugh C. A. (2015). *Leuconostoc* spp. Associated with root rot in sugar beet and their interaction with *Rhizoctonia solani*. *Phytopathology* 106 432–441. 26735061 10.1094/PHYTO-12-15-0325-R

[B101] Sun Z. B. Yuan X. F. Zhang H. Wu L. F. Liang C. Feng Y. J. (2013). Isolation, screening and identification of antagonistic downy mildew endophytic bacteria from cucumber. *Eur. J. Plant Pathol.* 137 847–857. 10.1007/s10658-013-0293-5

[B102] Truyens S. Weyens N. Cuypers A. Vangronsveld J. (2015). Bacterial seed endophytes: genera, vertical transmission and interaction with plants. *Environ. Microbiol. Rep.* 7 40–50. 10.1111/1758-2229.12181

[B103] Val-Moraes S. P. (2015). *Organic Amendments and Soil Suppressiveness in Plant Disease Management: Soil Biology*, eds Meghvansi M. K. Varma A. Cham: Springer, 175–184. 10.1007/978-3-319-23075-7

[B104] Verma S. K. Kingsley K. Irizarry I. Bergen M. Kharwar R. N. White J. F. (2017). Seed-vectored endophytic bacteria modulate development of rice seedlings. *J. Appl. Microbiol.* 122 1680–1691. 10.1111/jam.13463 28375579

[B105] Vespermann A. Kai M. Piechulla B. (2007). Rhizobacterial volatiles affect the growth of fungi and *Arabidopsis thaliana*. *Appl. Environ. Microbiol.* 73 5639–5641. 10.1128/AEM.01078-07 17601806 PMC2042089

[B106] Walterson A. M. Stavrinides J. (2015). *Pantoea*: insights into a highly versatile and diverse genus within the Enterobacteriaceae. *FEMS Microbiol. Rev.* 39 968–984. 10.1093/femsre/fuv027 26109597

[B107] Whipps J. M. (2001). Microbial interactions and biocontrol in the rhizosphere. *J. Exp. Bot.* 52 487–511. 10.1093/jexbot/52.suppl_1.487 11326055

[B108] Yang J. Kharbanda P. D. Mirza M. (2004). Evaluation of *Paenibacillus polymyxa* PKB1 for biocontrol of *Pythium* disease of cucumber in a hydroponic system. *Acta Hortic.* 635 59–66. 10.17660/ActaHortic.2004.635.7

