# Corrigendum: Bacterial Seed Endophytes of Domesticated Cucurbits Antagonize Fungal and Oomycete Pathogens Including Powdery Mildew

**DOI:** 10.3389/fmicb.2018.00995

**Published:** 2018-06-11

**Authors:** Eman M. Khalaf, Manish N. Raizada

**Affiliations:** ^1^Department of Plant Agriculture, University of Guelph, Guelph, ON, Canada; ^2^Department of Microbiology and Immunology, Faculty of Pharmacy, Damanhour University, Damanhour, Egypt

**Keywords:** seed endophytes, cucurbit, biocontrol, *Phytophthora capsici*, *Pythium aphanidermatum*, *Rhizoctonia solani*, *Fusarium graminearum*, powdery mildew

In the original article, there was an error. The species name *Pythium aphanidermatum* was misspelled as *aphanideratum*. Corrections have been made throughout the article, as well as in keywords, Figures 2, 3, and in Supplementary Table S1.

The authors apologize for this error and state that this does not change the scientific conclusions of the article in any way.

The original article has been updated.

## Conflict of interest statement

The authors declare that the research was conducted in the absence of any commercial or financial relationships that could be construed as a potential conflict of interest.

