# Supplementary material for: Bacterial Seed Endophytes of Domesticated Cucurbits Antagonize Fungal and Oomycete Pathogens Including Powdery Mildew
Source: Front Microbiol. 2018 Feb 5;9:42. doi: 10.3389/fmicb.2018.00042 (PMC5807410; doi:10.3389/fmicb.2018.00042)
Supplement: FIGURE S1 — Selected powdery mildew disease leaf photos corresponding to different levels of disease severity used as references to guide visual disease assessments of the detached whole leaf bioassay. (A–E) Shown are representative photos of tested cucumber leaves: (A) chemical fungicide positive control (Prothioconazole), (B) commercial biocontrol agent positive control (Bacillus subtilis strain QST 713), (C) example of a typical promising endophytic bacteria; Pantoea EKM103V, (D) negative control (LB broth amended with filter sterilized 0.01% Tween 20), (E) example of an inoculant Bacillus_EKM502B with adverse effect. R (1–5) denotes replicate number. [file Image_1.pdf]

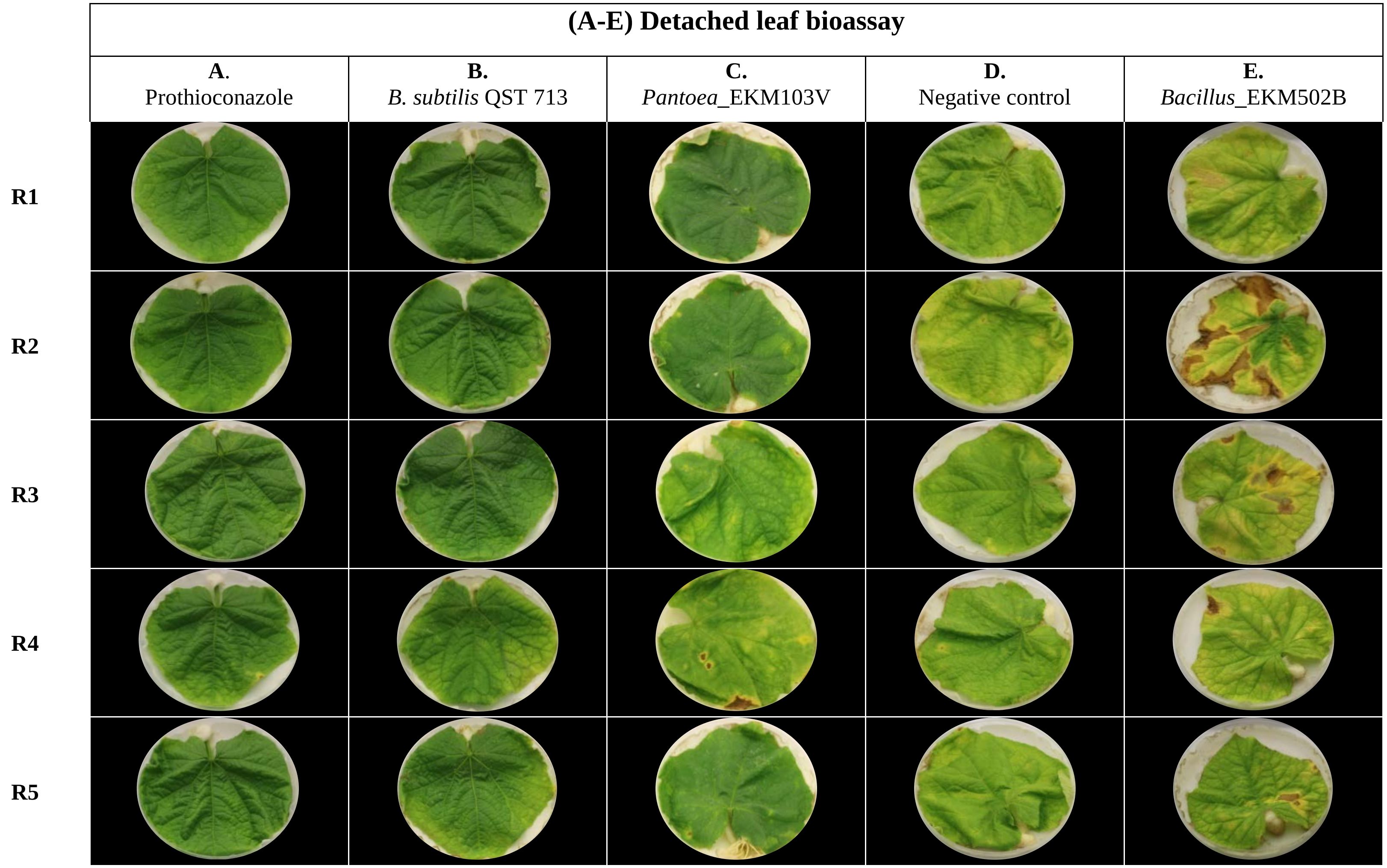

Figure S1. Selected powdery mildew disease leaf photos corresponding to different levels of disease severity used as references to guide visual disease assessments of the detached whole leaf bioassay. (A-E) Shown are representative photos of tested cucumber leaves: **(A)** chemical fungicide positive control (Prothioconazole), **(B)** commercial biocontrol agent positive control (*Bacillus subtilis* strain QST 713), **(C)** example of a typical promising endophytic bacteria; *Pantoea* EKM103V , **(D)** negative control (LB broth amended with filter sterilized 0.01% Tween 20), **(E)** example of an inoculant *Bacillus*_EKM502B with adverse effect. R (1-5) denotes replicate number.
