# Supplementary material for: Bacterial Seed Endophytes of Domesticated Cucurbits Antagonize Fungal and Oomycete Pathogens Including Powdery Mildew
Source: Front Microbiol. 2018 Feb 5;9:42. doi: 10.3389/fmicb.2018.00042 (PMC5807410; doi:10.3389/fmicb.2018.00042)
Supplement: TABLE S3 — Complete list of endophytic strains displaying a positive preventative effect (PE) against powdery mildew in the leaf disk bioassay, organized by bacterial phylum, genus and original seed source. Statistical analysis (Dunnett’s Multiple Comparison Test) of the disease index percentages (DI%) per treatment (n = 3) compared to the negative control is represented as calculated p values. A greater Disease Index indicates increased disease symptoms, whereas a greater Preventative Effect denotes improved control of the disease. The circumflex accent (ˆ) denotes that fresh fruits were used as the seed source. NS, non-significant, ∗p < 0.05, ∗∗p < 0.01, ∗∗∗p < 0.001, ∗∗∗∗p < 0.0001. [file Table_3.DOCX]

**Table S3. Complete list of endophytic strains displaying a positive preventative effect (PE) against powdery mildew in the leaf disc bioassay, organized by bacterial phylum, genus and original seed source.**

|  | **Isolate name** | **Host plant** | **Leaf disc bioassay** | | **Summary** | **P value** |
| --- | --- | --- | --- | --- | --- | --- |
| **Phylum** |  |  | **Disease index%** | **Preventive effect%** |  |  |
| Firmicutes | Bacillus_Strain_EKM111B | Cucumber (Marketmore) | 71.42 | 12.78 | NS | 0.9980 |
|  | Bacillus_Strain_EKM117B | Cucumber (Marketmore) | 80 | 2.31 | NS | 0.9997 |
|  | Bacillus_Strain_EKM119B | Cucumber (Marketmore) | 28.57 | 65.11 | **** | 0.0001 |
|  | Bacillus_Strain_EKM120B | Cucumber (Marketmore) | 61.9 | 24.41 | NS | 0.4165 |
|  | Bacillus_Strain_EKM122B | Cucumber (Marketmore) | 66.66 | 18.59 | NS | 0.8537 |
|  | Bacillus_Strain_EKM123B | Cucumber (Straight eight) | 70.47 | 13.94 | NS | 0.9844 |
|  | Bacillus_Strain_EKM124B | Cucumber (Straight eight) | 45.71 | 44.18 | ** | 0.0021 |
|  | Bacillus_Strain_EKM126B | Cucumber (Marketmore) | 26.66 | 67.43 | **** | 0.0001 |
|  | Bacillus_Strain_EKM127B | Cucumber (Straight eight) | 37.14 | 54.64 | **** | 0.0001 |
|  | Bacillus_Strain_EKM128B | Cucumber (Straight eight) | 73.33 | 10.45 | NS | 0.9985 |
|  | Bacillus_Strain_EKM201B | Melon (Cantaloupe delicious) | 42.85 | 47.67 | *** | 0.0006 |
|  | Bacillus_Strain_EKM202B |  | 55.23 | 32.55 | NS | 0.0765 |
|  | Bacillus_Strain_EKM210B | Melon^ (Honeydew) | 28.57 | 65.11 | **** | 0.0001 |
|  | Bacillus_Strain_EKM211B | Melon^ (Honeydew) | 28.57 | 65.11 | **** | 0.0001 |
|  | Bacillus_Strain_EKM215B | Melon^ (Santa Claus cantaloupe) | 32.38 | 60.46 | **** | 0.0001 |
|  | Bacillus_Strain_EKM306B | Pumpkin^ | 16.19 | 80.23 | **** | 0.0001 |
|  | Bacillus_Strain_EKM408B | Watermelon (Crimson sweet) | 42.85 | 47.67 | *** | 0.0006 |
|  | Bacillus_Strain_EKM409B | Watermelon (Crimson sweet) | 64.76 | 20.92 | NS | 0.6821 |
|  | Bacillus_Strain_EKM410B | Watermelon (Crimson sweet) | 27.61 | 66.27 | **** | 0.0001 |
|  | Bacillus_Strain_EKM411B | Watermelon (Early Canada Improved) | 46.66 | 43.01 | ** | 0.0033 |
|  | Bacillus_Strain_EKM417B | Watermelon (Crimson sweet) | 21.9 | 73.25 | **** | 0.0001 |
|  | Bacillus_Strain_EKM418B | Watermelon^ | 38.09 | 53.48 | **** | 0.0001 |
|  | Bacillus_Strain_EKM419B | Watermelon^ | 8.57 | 89.53 | **** | 0.0001 |
|  | Bacillus_Strain_EKM420B | Watermelon (Crimson sweet) | 48.57 | 40.69 | ** | 0.0072 |
|  | Bacillus_Strain_EKM502B | Butternut^ | 26.66 | 67.43 | **** | 0.0001 |
|  | Bacillus_Strain_EKM503B | Acorn squash^ | 27.61 | 66.27 | **** | 0.0001 |
|  | Bacillus_Strain_EKM504B | Spaghetti squash^ | 42.85 | 47.67 | *** | 0.0006 |
|  | Bacillus_Strain_EKM505B | Spaghetti squash^ | 23.8 | 70.92 | **** | 0.0001 |
|  | Bacillus_Strain_EKM702B | Bottle gourd | 24.76 | 69.76 | **** | 0.0001 |
|  | Bacillus_Strain_EKM703B | Bottle gourd | 20.95 | 74.41 | **** | 0.0001 |
|  | Bacillus_Strain_EKM704B | Bottle gourd | 77.14 | 5.80 | NS | 0.9993 |
|  | Bacillus_Strain_EKM705B | Bottle gourd | 72.38 | 11.62 | NS | 0.9983 |
|  | Bacillus_Strain_EKM706B | Bottle gourd | 40 | 51.15 | *** | 0.0002 |
|  | Paenibacillus_Strain_EKM103P | Cucumber (Burpless Beauty) | 40 | 51.15 | *** | 0.0002 |
|  | Paenibacillus_Strain_EKM203P | Melon (Cantaloupe delicious) | 59.04 | 27.90 | NS | 0.2198 |
|  | Paenibacillus_Strain_EKM208P | Melon (Cantaloupe delicious) | 74.28 | 9.29 | NS | 0.9987 |
|  | Paenibacillus_Strain_EKM210P | Melon (Cantaloupe delicious) | 33.33 | 59.29 | **** | 0.0001 |
|  | Paenibacillus_Strain_EKM303P | Pumpkin (Jack O’lantern) | 26.66 | 67.43 | **** | 0.0001 |
|  | Paenibacillus_Strain_EKM305P | Pumpkin^ | 27.61 | 66.27 | **** | 0.0001 |
|  | Paenibacillus_Strain_EKM306P | Pumpkin^ | 20.95 | 74.41 | **** | 0.0001 |
|  | Paenibacillus_Strain_EKM307P | Pumpkin^ | 45.71 | 44.18 | ** | 0.0021 |
|  | Paenibacillus_Strain_EKM308P | Pumpkin^ | 15.23 | 81.39 | **** | 0.0001 |
|  | Paenibacillus_Strain_EKM309P | Pumpkin^ | 40 | 51.15 | *** | 0.0002 |
|  | Paenibacillus_Strain_EKM310P | Pumpkin^ | 29.52 | 63.95 | **** | 0.0001 |
|  | Paenibacillus_Strain_EKM312P | Pumpkin^ | 10.47 | 87.20 | **** | 0.0001 |
|  | UnclassifiedPaenibacillaceae_  Strain_EKM501M | Spaghetti squash^ | 19.04 | 76.74 | **** | 0.0001 |
|  | Lactococcus_Strain_EKM102L | Cucumber (Straight eight) | 24.76 | 69.76 | **** | 0.0001 |
|  | Lactococcus_Strain_EKM201L | Melon^ (Cantaloupe) | 62.85 | 23.25 | NS | 0.4998 |
|  | Lactococcus_Strain_EKM202L | Melon^ (Cantaloupe) | 71.42 | 12.78 | NS | 0.9980 |
|  | Lactococcus_Strain_EKM203L | Melon^ (Cantaloupe) | 20.95 | 74.41 | **** | 0.0001 |
|  | Lactococcus_Strain_EKM501L | Acorn squash^ | 10.47 | 87.20 | **** | 0.0001 |
|  | Lactococcus_Strain_EKM502L | Acorn squash^ | 18.09 | 77.90 | **** | 0.0001 |
|  | Pediococcus_Strain_EKM201D | Melon^ (Cantaloupe) | 14.28 | 82.55 | **** | 0.0001 |
|  | Pediococcus_Strain_EKM202D | Melon^ (Cantaloupe) | 8.57 | 89.53 | **** | 0.0001 |
|  | Staphylococcus_Strain_EKM201S | Melon^ (Honeydew) | 19.04 | 76.74 | **** | 0.0001 |
| Proteobacteria | UnclassifiedEnterobacteriaceae_  Strain_EKM102E | Cucumber (Spacemaster) | 13.33 | 83.71 | **** | 0.0001 |
|  | UnclassifiedEnterobacteriaceae_  Strain_EKM103E | Cucumber^ (seed wash) | 80.95 | 1.15 | NS | 0.9999 |
|  | UnclassifiedEnterobacteriaceae_  Strain_EKM102Z | Cucumber^ (seed wash) | 24.76 | 69.76 | **** | 0.0001 |
|  | UnclassifiedEnterobacteriaceae_  Strain_EKM301K | Pumpkin^ | 11.42 | 86.04 | **** | 0.0001 |
|  | UnclassifiedEnterobacteriaceae_  Strain_EKM302K | Pumpkin^ | 20 | 75.57 | **** | 0.0001 |
|  | UnclassifiedEnterobacteriaceae_  Strain_EKM301Z | Pumpkin^ | 12.38 | 84.88 | **** | 0.0001 |
|  | UnclassifiedEnterobacteriaceae_  Strain_EKM403G | Watermelon (Crimson sweet) | 39.04 | 52.32 | *** | 0.0001 |
|  | UnclassifiedEnterobacteriaceae_  Strain_EKM601Z | Chinese okra | 41.9 | 48.83 | *** | 0.0004 |
|  | UnclassifiedEnterobacteriaceae_  Strain_EKM602Z | Chinese okra | 17.14 | 79.06 | **** | 0.0001 |
|  | UnclassifiedEnterobacteriaceae_  Strain_EKM603Z | Chinese okra | 37.14 | 54.64 | **** | 0.0001 |
|  | Pantoea_Strain_EKM101V | Cucumber^ (seed wash) | 9.52 | 88.37 | **** | 0.0001 |
|  | Pantoea_Strain_EKM103V | Cucumber^ (seed wash) | 21.9 | 73.25 | **** | 0.0001 |
|  | Cronobacter_Strain_EKM102R | Cucumber (Spacemaster) | 43.8 | 46.50 | *** | 0.0009 |
|  | Pseudomonas_Strain_EKM103A | Cucumber^ (seed wash) | 28.57 | 65.11 | **** | 0.0001 |
|  | Pseudomonas_Strain_EKM402A | Watermelon^ | 31.42 | 61.62 | **** | 0.0001 |
|  | Pseudomonas_Strain_EKM403A | Watermelon^ | 10.47 | 87.20 | **** | 0.0001 |
| Actino-  bacteria | Microbacterium_Strain_EKM101T | Cucumber^ | 26.66 | 67.43 | **** | 0.0001 |
|  | Microbacterium_Strain_EKM102T | Cucumber^ | 21.9 | 73.25 | **** | 0.0001 |
|  | UnclassifiedMicrobacteriaceae_  Strain_EKM601Y | Chinese okra | 32.38 | 60.46 | **** | 0.0001 |
|  | Rathayibacter_Strain_EKM201Y | Melon^ (Canary cantaloupe) | 43.8 | 46.50 | *** | 0.0009 |
|  | Micrococcus_Strain_EKM201U | Melon^ (Santa Claus cantaloupe) | 33.33 | 59.29 | **** | 0.0001 |
|  | Micrococcus_Strain_EKM501U | Acorn squash^ | 26.66 | 67.43 | **** | 0.0001 |
| Controls | Prothioconazole | Chemical fungicide | 31.42 | 61.62 | **** | 0.0001 |
|  | Bacillus subtilis strain QST 713 | Biofungicide | 39.04 | 52.32 | **** | 0.0001 |
|  | Negative control | - | 81.90 | - |  |  |

^: fresh fruits were used as a source of seeds

NS: Non-significant, *: p < 0.05, **: p< 0.01, ***: p < 0.001, ****: p < 0.0001
